# Protein-modified nanomaterials: emerging trends in skin wound healing

**DOI:** 10.1186/s11671-023-03903-8

**Published:** 2023-10-16

**Authors:** Deepinder Sharda, Pawandeep Kaur, Diptiman Choudhury

**Affiliations:** 1 School of Chemistry and Biochemistry, Thapar Institute of Engineering and Technology, Patiala, Punjab 147004 India; 2 Thapar Institute of Engineering and Technology-Virginia Tech Centre of Excellence for Emerging Materials, Thapar Institute of Engineering and Technology, Patiala, Punjab 147004 India

**Keywords:** Wound healing, Nanoformulations, Growth factors, Proteins, Antidiabetic agents

## Abstract

Prolonged inflammation can impede wound healing, which is regulated by several proteins and cytokines, including IL-4, IL-10, IL-13, and TGF-β. Concentration-dependent effects of these molecules at the target site have been investigated by researchers to develop them as wound-healing agents by regulating signaling strength. Nanotechnology has provided a promising approach to achieve tissue-targeted delivery and increased effective concentration by developing protein-functionalized nanoparticles with growth factors (EGF, IGF, FGF, PDGF, TGF-β, TNF-α, and VEGF), antidiabetic wound-healing agents (insulin), and extracellular proteins (keratin, heparin, and silk fibroin). These molecules play critical roles in promoting cell proliferation, migration, ECM production, angiogenesis, and inflammation regulation. Therefore, protein-functionalized nanoparticles have emerged as a potential strategy for improving wound healing in delayed or impaired healing cases. This review summarizes the preparation and applications of these nanoparticles for normal or diabetic wound healing and highlights their potential to enhance wound healing.

**Graphical abstract:**

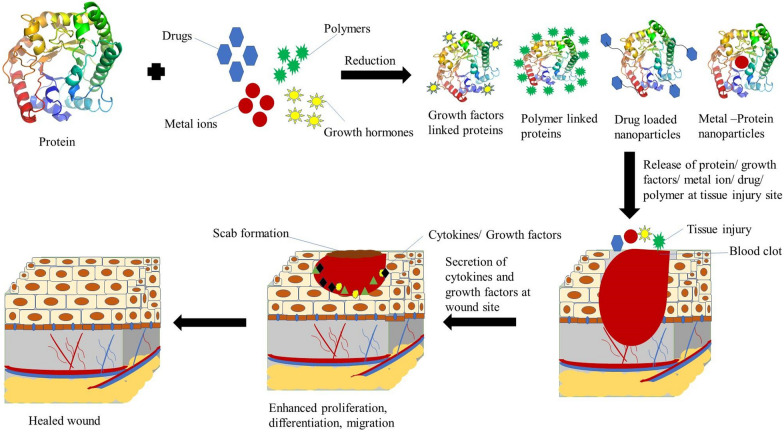

## Introduction

Nanomaterials have high drug-loading efficacy, which can be attributed to a high surface area-to-volume ratio. They respond quickly to any minute alteration in the surrounding environment, like a magnetic field, pH, and temperature [1]. The bionanomaterials like peptides, biomolecules, enzymes, and protein-functionalized formulations have various biological applications ranging from bioimaging [2], catalysis [3], fluorescent biolabeling [4, 5] hyperthermia [6, 7] tissue engineering [8], gene and drug delivery [9, 10], and so on. Moreover, protein-functionalized and stabilized nanomaterials exhibit numerous features such as sensing, biocompatibility, plasmon-enhanced catalysis, targeted nanocarriers, and drug delivery [11, 12]. The constituting units of the proteins behave not only as reducing and chelating agents that help in developing nanoclusters but also allow crystalline [13, 14] and amorphous [15] growth of the nanostructures of different sizes and shapes [16]. The target-specific binding ability of proteins enhances their action efficiency and helps to cure the wound [15, 17]. However, the poor permeability through membranes, short half-life, and high enzymatic degradation risk pose a serious issue to the targeted delivery of potent therapeutic proteins to the site and thus require slight modifications for efficient delivery [18, 19]. Protein-functionalized nanoformulations, such as protein-capped metal nanoparticles, protein-encapsulated nanostructures, and protein nanocarriers, including hydrogels, scaffolds, liposomes, nanotubes, nanogels, nanoparticles, polymeric particles and poly(ester amide) PEA (synthesized using amino acids, diacids, and diols), were developed to effectively deliver the protein/drug at the effected/targeted site [20, 21].

The skin is the largest human body organ that, by acting as a barrier, assists in preventing the entry of harmful microbes into the body. The skin consists mainly of epidermal, dermal, and hypodermal layer, which contains certain hormonal glands, hair, and nerve endings that make the skin a complex organ. It has the ability to self-healing to a certain extent which is impaired in case of extensive damage caused by chemical or physical shock, making the healing a challenging process [22]. Wound healing is a cumbersome process that activates a series of physiological and simultaneous phases such as hemostasis, inflammation, proliferation, and remodeling [23, 24], which have a significant effect on the effective wound or infected area treatment. The classical process of healing begins with the hemostasis, which includes clot formation due to the activation of platelets that release the chemokines and growth factors (including fibroblasts and keratinocytes) at the wound site and acts as key parameters of hemostasis and coagulation. They further assist in preventing the entry of bacteria at the affected site and regulate antimicrobial peptide production by expressing distinct toll-like receptors (TLR) [25, 26]. After this inflammatory phase begins, which is quite complex due to the extrinsic and intrinsic factors and the excessive and limited, both inflammation conditions delay the wound healing and promote injury. It acts as the basic defense system against pathogenic invasion and is started in response to signals induced by injury, the release of damage-associated molecular patterns, and pathogen-associated molecular patterns by necrotic cells and damaged tissue and bacterial components, respectively. Vasodilation is promoted by pro-inflammatory cytokines, and these proinflammatory signals and activated signaling pathways stimulate the secretion of cytokines by neutrophils. Neutrophils, by phagocytosis, remove the necrotic tissue and pathogens and cause the secretion of antimicrobial peptides, proteolytic enzymes, and eicosanoids [27, 28]. Following this, the proliferative phase begins, in which there is the accumulation of cells and connective tissues along with the activation of numerous factors, including fibroblasts, keratinocytes, macrophages, angiogenesis factors, and endothelial cells across the wound site. Keratinocytes are responsible for regenerating the epidermis by differentiation and basement membrane reformation. Fibroblasts play a role in developing the matrix consisting of granulation tissue which comprises fibronectin, proteoglycans, and immature collagen, which collectively acts as a scaffold essential for cell adhesion and migration across the wound [29, 30]. During angiogenesis, new blood vessels are generated in order to fulfill the demand for highly proliferative regenerative tissue, and macrophages assist in guiding vessel tips together and eradicating the superfluous vessels by phagocytosis for making new vasculature critical for transportation across the site [31, 32]. In the last phase of remodeling, there occurs the resynthesis of the extracellular matrix for maintaining the balance between the death of old cells and the formation of new cells. Fibroblasts are the major cells crucial for remodeling by replacing the fibrin clot with fibronectin, proteoglycan, and collagen fibril synthesis. Collagen III is finally replaced with collagen I, which enhances the tensile strength of wound scar and helps wound closure at a faster pace [33–35]. In the initial healing stages, there occur the release of cytokines and activation of leukocytes by macrophages in order to exhibit a pro-inflammatory environment [36]. The potent release of anti-inflammatory cytokines, insulin-like growth factor (IGF), and proteins such as insulin are crucial for aiding in efficient wound healing, which is initiated by inducing angiogenesis and re-epithelialization [37]. The proteins (insulin, collagen, keratin, gelatin), growth factors such as insulin-like growth factors (IGF), epidermal growth factors (EFG), fibroblast growth factors (FGF), platelet-derived growth factor (PDGF), transforming growth factor-beta 1 (TGF-β1), tumor necrosis factor-alpha (TNF-α), and vascular endothelial growth factor (VEGF), etc., help in reducing inflammation, cell proliferation, and remodeling [38, 39]. The growth factors act as the endothelial signaling factors which assists in regulating the cellular processes during wound healing. There are numerous studies investigating the critical role played by them in modulating the healing effects in normal and diabetic conditions. The treatment of wounds at a faster pace is highly dependent upon the transition of cytokines from pro-inflammatory to anti-inflammatory ones [40]. In chronic wounds, a halt in the conversion of cytokines from one form to another causes a prolonged pro-inflammatory phase which eventually results in delayed wound healing [41].

**Fig. 1:**
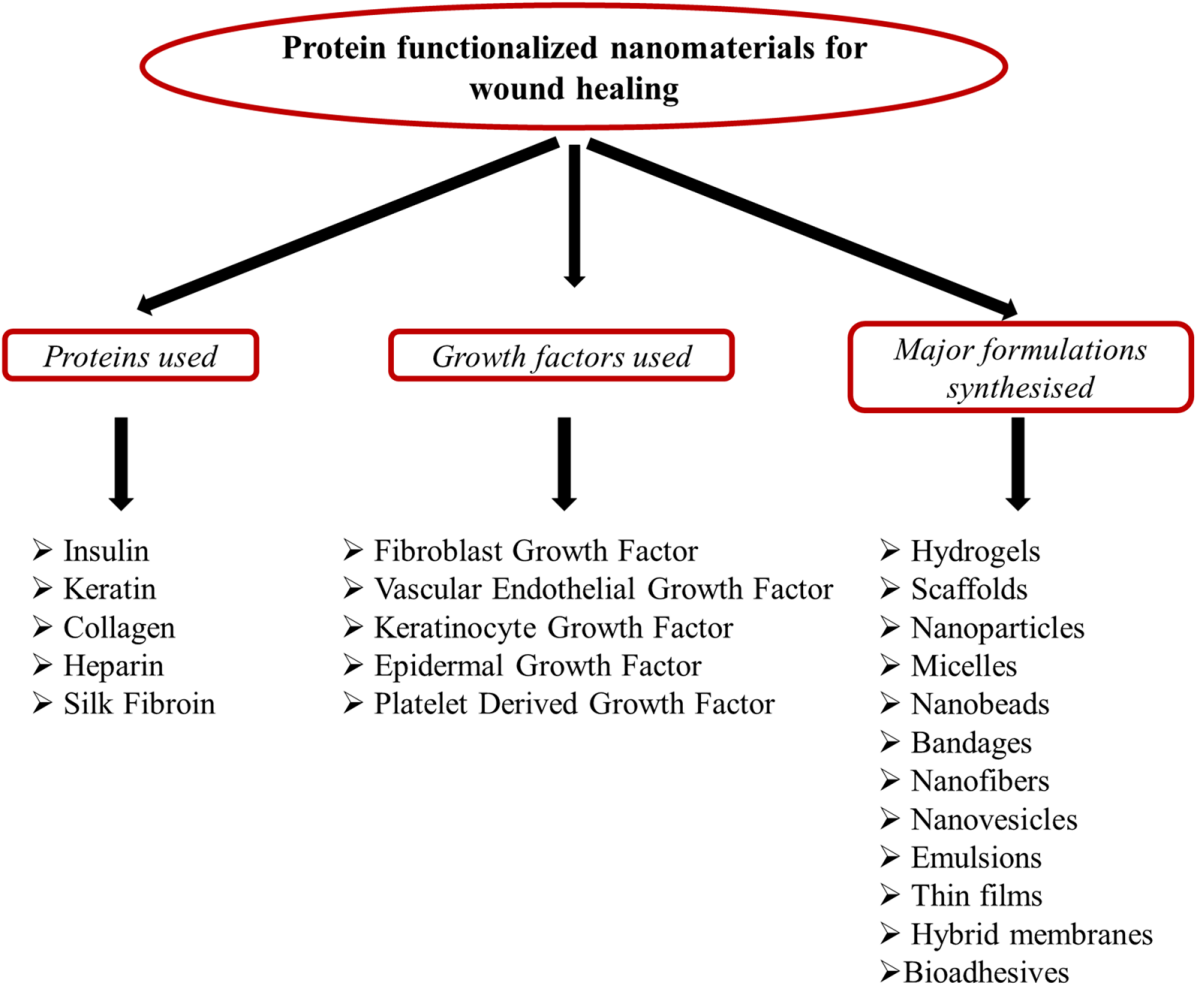
The figure shows different proteins and growth factors widely used for making different types of nanoformulations for wound healing and skin regeneration activity

Depending upon these protein and growth factor features and their role in normal and diabetic wound healing, there is an increasing demand for the development of reliable and cost-effective wound dressings, and protein-functionalized nanoformulations are the ones that have high wound-healing potency, target specificity, and are easy to synthesize using green methods. The significance of this field is very diverse as the treatment of chronic wounds is becoming difficult day by day due to the development of antibiotic resistance against the available medications, degradation of formulations under unfavorable conditions, uncontrolled drug release, and less target specificity and efficiency. The cost of healing ranges fall between $28.1 billion and $96.8 billion for acute and chronic wounds, and the maximum amount is used for surgical wounds, followed by diabetic ulcers [42]. The increasing prevalence of chronic wounds, the growing geriatric population, and the rising number of surgeries are some factors driving the growth of the wound care market. The number of patients affected by chronic wounds is approximately 5.7 million people in the USA alone, and an estimated cost of USD 25 billion is spent per year [43]. Recent advancements in bionanomaterials have led researchers toward developing optimized protein-based nanoformulations for wound-healing purposes as they showed sustained drug release, reduced administration frequency, an adequate concentration of medicine for an extended period, high efficiency in wound healing compared to the free proteins, high protein stability, easy transport through the body and less denaturation under environmental conditions, all work together to form advanced formulations critical for wound healing. The high surface area to volume ratio, water solubility, stability, biocompatibility, target specificity, and biodegradability have given an upper hand to the use of nanoformulations over traditional therapeutics. All these factors have significantly contributed to gaining the enormous interest of researchers in this desirable field for developing and exploring better futuristic approaches in synthesizing protein-functionalized materials [44]. Figure 1 shows various proteins and growth factors primarily used in wound healing along with the major nanoformulations being developed using them.

This review article focuses on protein-functionalized nanomaterials for wound-healing applications. The key terms covered in this article include protein-functionalized nanoparticles, bionanomaterials, drug delivery, tissue engineering, gene delivery, cytokines, growth factors, inflammation, macrophages, angiogenesis, and skin regeneration. Other terms discussed include bioimaging, catalysis, fluorescent biolabeling, hyperthermia, plasmon-enhanced catalysis, and targeted nanocarriers. The article highlights the advantages of protein-functionalized nanomaterials over only protein, including their high drug-loading efficacy, quick response to environmental changes, and target-specific binding ability. This review demonstrates the enormous possibilities of developing green and biocompatible protein nanoformulations with high efficacy and specificity for wound-healing applications.

## Structural details of major proteins and growth factors and their potential role in skin wound healing

Several proteins are involved in wound healing, including antidiabetic proteins such as insulin or extracellular matrix proteins such as heparin, keratin, silk fibroin, and collagen. We will discuss them briefly in the coming sections.

### Insulin

**Fig. 2:**
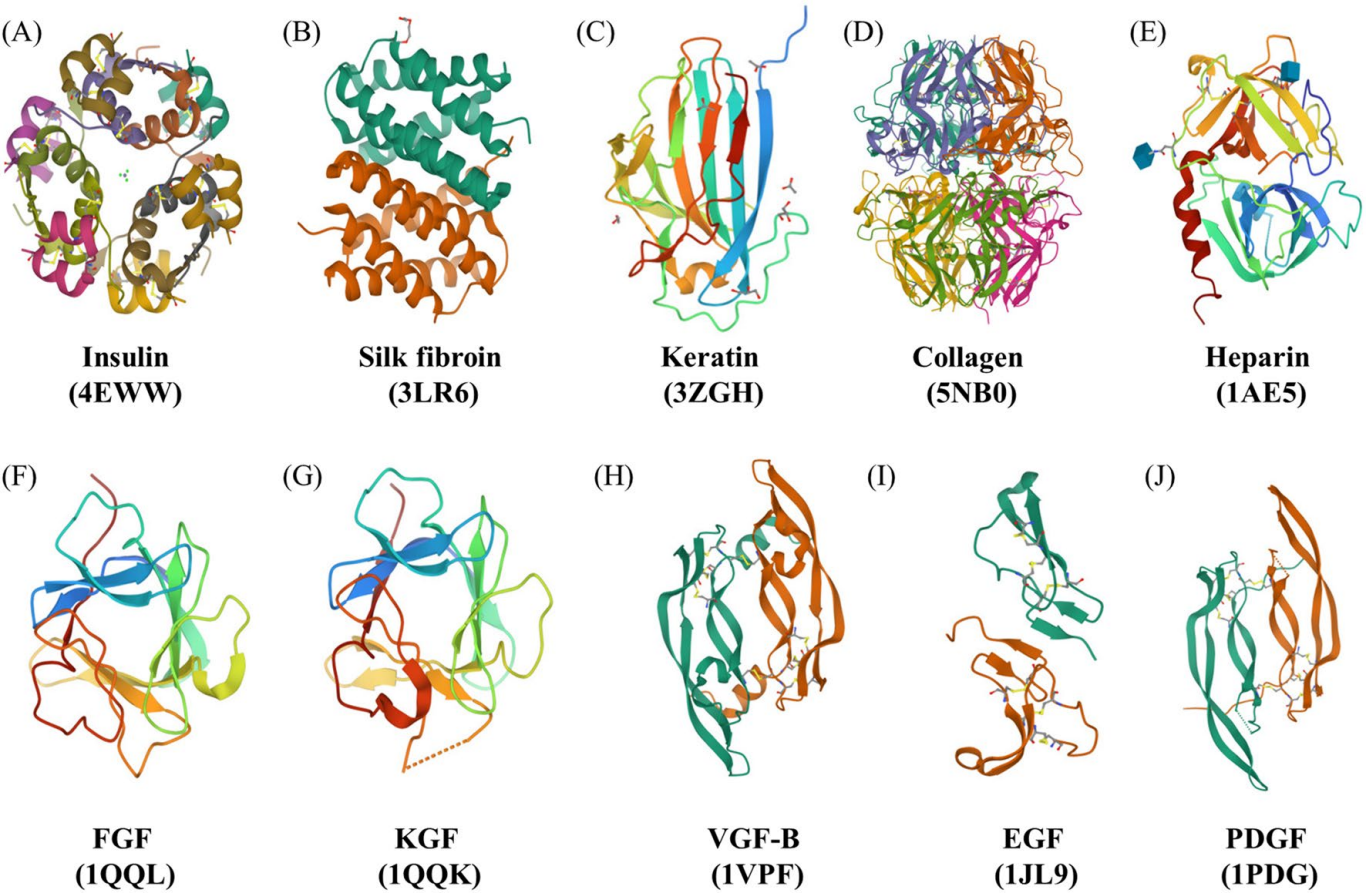
The crystal structures of different proteins and growth factors along with their PDB IDs **a** insulin, **b** silk fibroin, **c** keratin, **d** collagen, and **e** heparin, **f** fibroblast growth factor (FGF), **g** keratinocyte growth factor (KGF), **h** vascular growth factor (VGF-B), **i** epidermal growth factor (EGF), and **j** platelets-derived growth factor (PDGF)

**Fig. 3:**
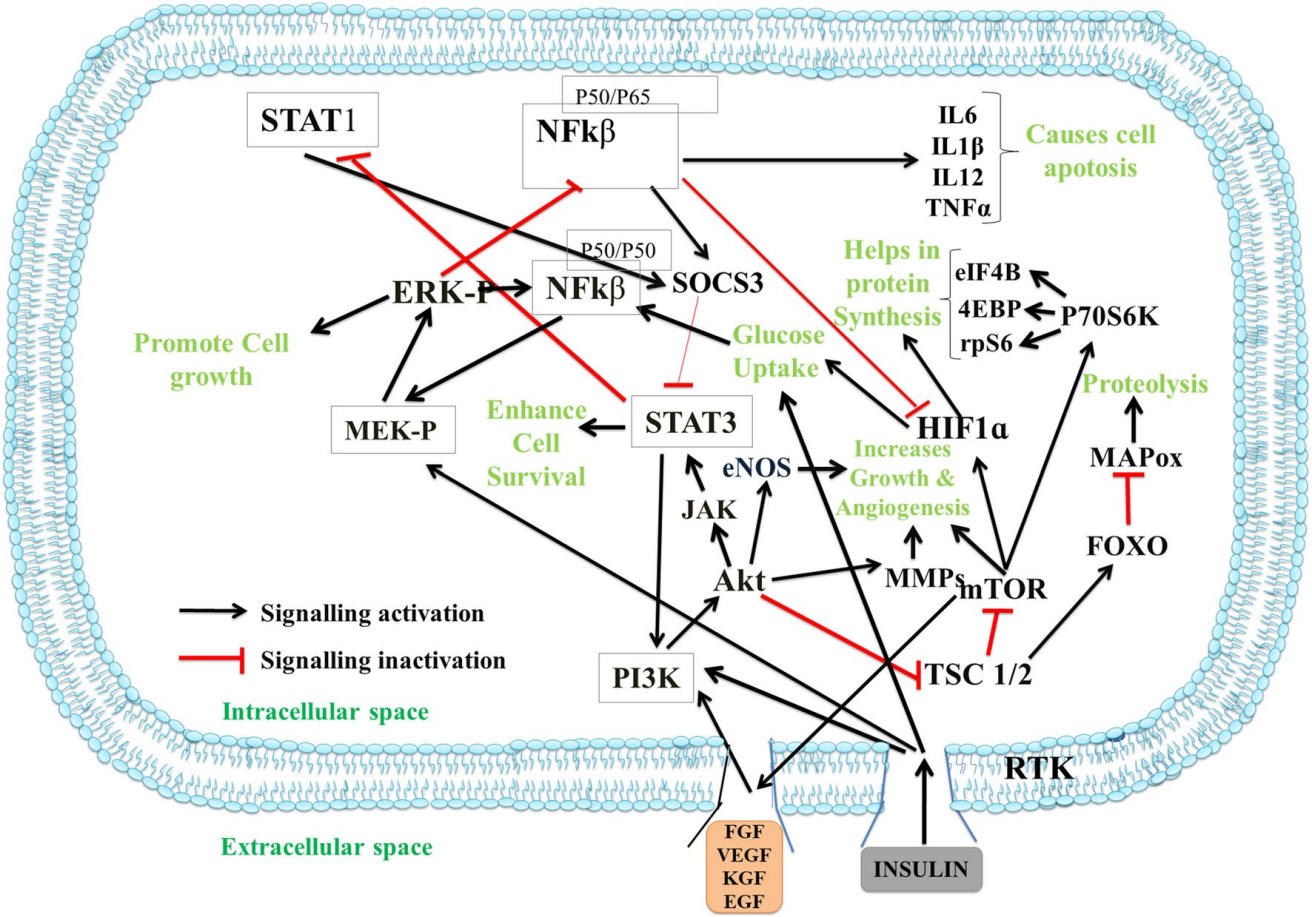
The figure shows the signaling pathway followed by insulin and other growth factors for wound recovery. The generation of IFN-γ and TNF-α activated the STAT-1, IRF-3, and NF-kβ, which are responsible for the secretion of IL-10, IL-12, and other interleukins for the transition of pro-inflammatory cytokines to anti-inflammatory ones and promotes healing

It is derived from its inactive form, proinsulin, in which the N-terminal of chain A is connected with the C-terminal of chain B through a C-peptide which is removed during the maturation. Insulin is a peptide hormone composed of two polypeptide chains, chain A and chain B having a total of 51 amino acids, of which 17 are proteinogenic amino acids. The two chains are interconnected via three disulfide bridges, and its folding occurs with three alpha-helices (two in chain A and 1 in chain B) and a small β-sheet segment. The presence of zinc ions and phenolic ligands helps in the dimerization of insulin and further promotes the formation of hexamers (Fig. 2A) The molecular weight of insulin is around 5 kDa. Depending upon its structure and low molecular weight, it is widely employed for synthesizing nanoformulations that are biocompatible, non-toxic, and can be easily inserted into the body or topically applied over the wound and hence promote wound healing by following critical mechanisms. It regulates blood glucose levels by stimulating glucose uptake in cells. It also plays essential roles in cellular differentiation, lipid and protein biosynthesis, growth factor activity, and wound healing. It also acts as a growth factor and increases the migration ability of cells, thereby aiding in wound healing [45, 46]. The insulin receptor is related to receptor tyrosine kinase transmembrane signaling proteins present on the surface of cells. The Akt and Erk signaling pathways are activated upon binding, improving wound healing [47, 48]. Insulin induces the synthesis of proteins by the PI3K and Akt pathway, which helps form 4EBPI and ribosomal protein S6 essential for cell survival. It also binds to IGF receptors and exhibits anti-inflammatory activity through different signaling pathways, such as Akt and PI3K, which activate pro-inflammatory cytokine STAT-3, promoting cell growth and angiogenesis [39]. In addition, insulin inactivates the TNF-α-mediated inflammatory pathway, which inactivates the pro-inflammatory cytokines [39]. Insulin enhances the proliferation, migration, and secretion of different cells, including keratinocytes, fibroblasts, and endothelial cells [49]. Hence, it is used in integration with other wound dressings due to its low cost compared with other growth regulators [46]. Insulin signaling in wound healing plays a crucial role in cell proliferation, migration, and angiogenesis, making it an essential peptide hormone for wound healing as shown in Fig. 3.

### Silk fibroin

**Fig. 4:**
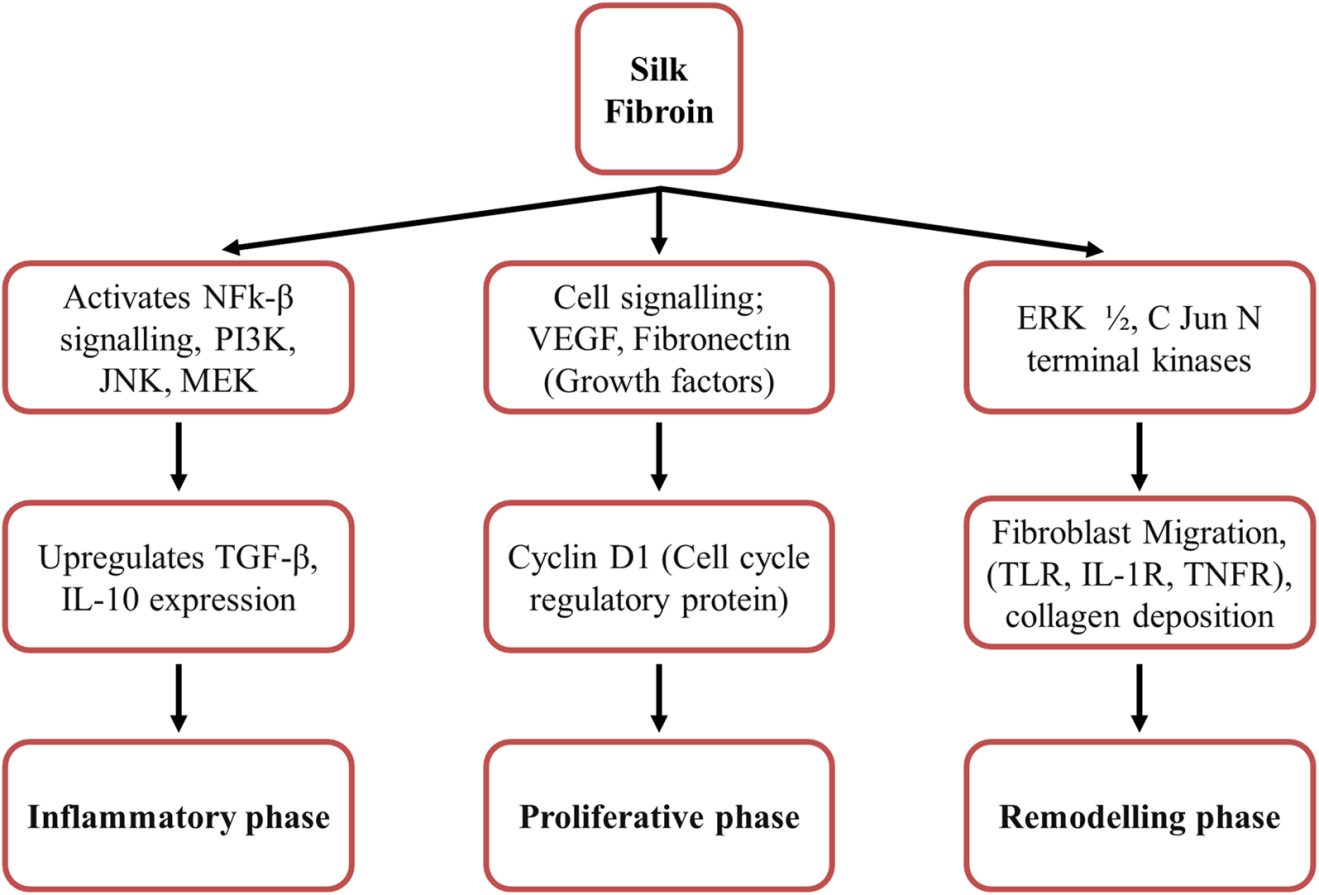
It shows the potential role and signaling pathway followed by silk fibroin in wound-healing activity by modulating inflammatory, proliferative, and remodeling phase of healing

Silk fibroin, derived from Bombyx mori, is a natural fibrous protein that acts as an essential biomaterial for tissue engineering and regeneration [50]. The silk fibroin is the main structural protein of silk and consists of a polypeptide chain with molecular weight in the range of 200–350 kDa. The repetitive units of hydrophobic heavy chains (391.6 kDa) and hydrophilic light chains (27.7 kDa) with terminal N and C groups constitute the primary silk fibroin structure, and the disulfide bonds connect the two chains together. Glycoprotein P25 provides integrity to the above chains, and the molar ratio of heavy chain, light chain, and P25 is 6:6:1 (Fig. 2B). The amino acids present in the hydrophobic chain include 45.9% glycine (Gly), 30.3% alanine (Ala), 12.1% serine (Ser), 5.3% tyrosine (Tyr), and 1.8% valine (Val), while the hydrophilic chain consists of 14% alanine (Ala), 10% serine (Ser), 9% glycine (Gly), and acetylated N-terminal Ser residues [51]. The structure of silk fibroin is such that it acts as a tissue scaffold or mesh for the attachment of growing cells and promotes functional tissue regeneration crucial for healing. Further, the β-sheet pattern is high in silk fibroin which promotes cell adhesion and differentiation in mesenchymal stem cells. It supports the proliferation, differentiation, and adhesion of various cells, including keratinocytes, endothelial cells, epithelial cells, fibroblasts, and osteoblasts, promoting wound healing [52, 53]. In addition to its role as a biomaterial, silk fibroin also regulates different signaling pathways crucial for wound healing. The NF-ĸB pathway is activated by silk fibroin, resulting in the upregulation of genes involved in cell proliferation, migration, and angiogenesis. Silk fibroin also enhances the activation of the ERK1/2 and Akt signaling pathways, leading to increased cell proliferation, migration, and extracellular matrix synthesis [54]. Due to its biocompatibility, non-toxicity, non-carcinogenicity, and less immunogenicity, silk fibroin is extensively studied in various biomedical and biological areas, and its ability to regulate different signaling pathways and support various cell functions makes it a promising biomaterial for wound-healing applications [34, 55] (Fig. 4).

### Keratin

**Fig. 5:**
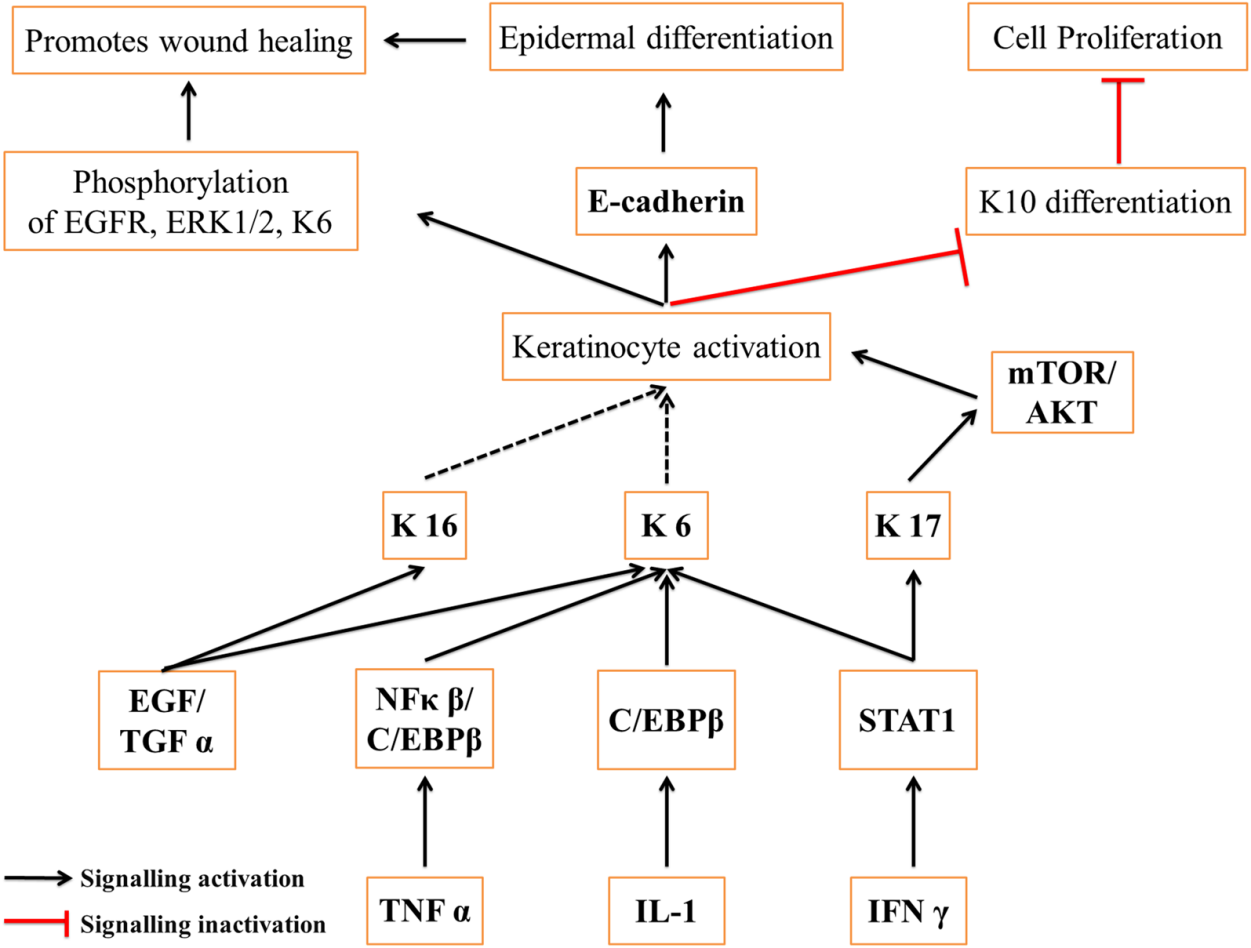
The figure demonstrates the signaling pathway followed by the keratin protein in wound healing. The three significant keratins, K6, K16, and K17, get secreted in response to injury and activate the keratinocytes, further promoting epidermal regeneration and wound healing

Keratin is a natural fibrous protein found in both humans and animals [56]. It is a scleroprotein made up of different amino acid residues but mainly rich in cysteine, and these amino acids are connected by intra- and intermolecular hydrogen bonds, disulfide bonds, ionic bonds, and hydrophobic interactions. Generally, it is found in two forms: alpha keratins, in which polypeptide chains are arranged in the form of alpha helices, and filaments have a diameter of 7–10 nm, whereas the beta keratins comprise beta sheets which are of 3–4 nm diameter (Fig. 2C). The alpha form is dominant in hairs, nails, horns, wool, and stratum corneum, whereas the beta form is found in feathers, reptilian claws, and scales [57]. Based on the structure, keratin is used for making scaffolds as it contains both alpha and beta forms which help in cell adhesion to the scaffold and enhances their proliferation and differentiation. Further, the presence of cell binding motifs, including leucine-aspartic acid-valine and glutamic acid-aspartic acid-serine, promotes the ability to promote cellular attachment. In various forms, such as hydrogels, scaffolds, and films, it has been widely used for bone regeneration, nerve regeneration, cell culture, and wound healing [58]. Keratin supports wound healing by accelerating hemostasis, promoting cell growth, and upregulating the expression of proteins relevant to wound healing. Additionally, it enhances plasma coagulation and lateral growth of fibrils [59, 60]. Previous studies have shown that keratin can arrest hemorrhage in bleeding animals, increase fibroblast proliferation and attachment, and upregulate the expression of keratinocytes involved in migration and collagen deposition [61, 62]. The mechanism underlying keratin-mediated wound healing is complex. TNF-α activates the NFκβ/C/EBPβ, and IL-1 activates the C/EBPβ, which in turn activates the K6 keratin. IFN-γ upregulates STAT1, activating the k6 and k17 keratins. Similarly, the k16 and k6 get activated by EGF/TGF α. All three keratins, K6, K16, and K17, activate the keratinocytes and promote E-cadherin secretion or phosphorylation of EGFR, ERK1/2, and K6, which increases epidermal differentiation and ultimately enables wound healing [63, 64] as shown in Fig. 5.

### Collagen

Collagen is a fibrous protein that provides structural support to the extracellular matrix, essential for tissue repair and regeneration. Skin mainly consists of collagen I. Collagen fiber has a coiled structure, and each fiber is up to 3 μm in diameter. Each fiber further consists of bundles of small fibrils, and the diameter of each fibril is 10–300 nm in diameter and several micrometers long. Fibrils are made up of triple-stranded collagen molecules. Based on this triple helix structure, the strands are interwoven together and allow the robust structure to maintain itself in tissues for years due to its stereo-dynamic stability as given in Fig. 2D [65]. Due to its fiber-like nature, it is widely employed for making scaffolds that have the potency of enhanced cellular attachment and thus promote wound-healing activity by providing space for cell growth and differentiation. Collagen-based biomaterials have been extensively studied for their wound-healing properties due to their ability to promote healing by enhancing cell proliferation, angiogenesis, and collagen deposition [66, 67]. Collagen stimulates the expression of growth factors such as TGF-β, activates the MAPK/ERK signaling pathway, and modulates the activity of enzymes involved in wound healing, such as MMPs [68]. Collagen-based biomaterials have shown great potential for wound-healing applications due to their biocompatibility, biodegradability, and ability to support cell growth and tissue regeneration [69].

### Heparin

**Fig. 6:**
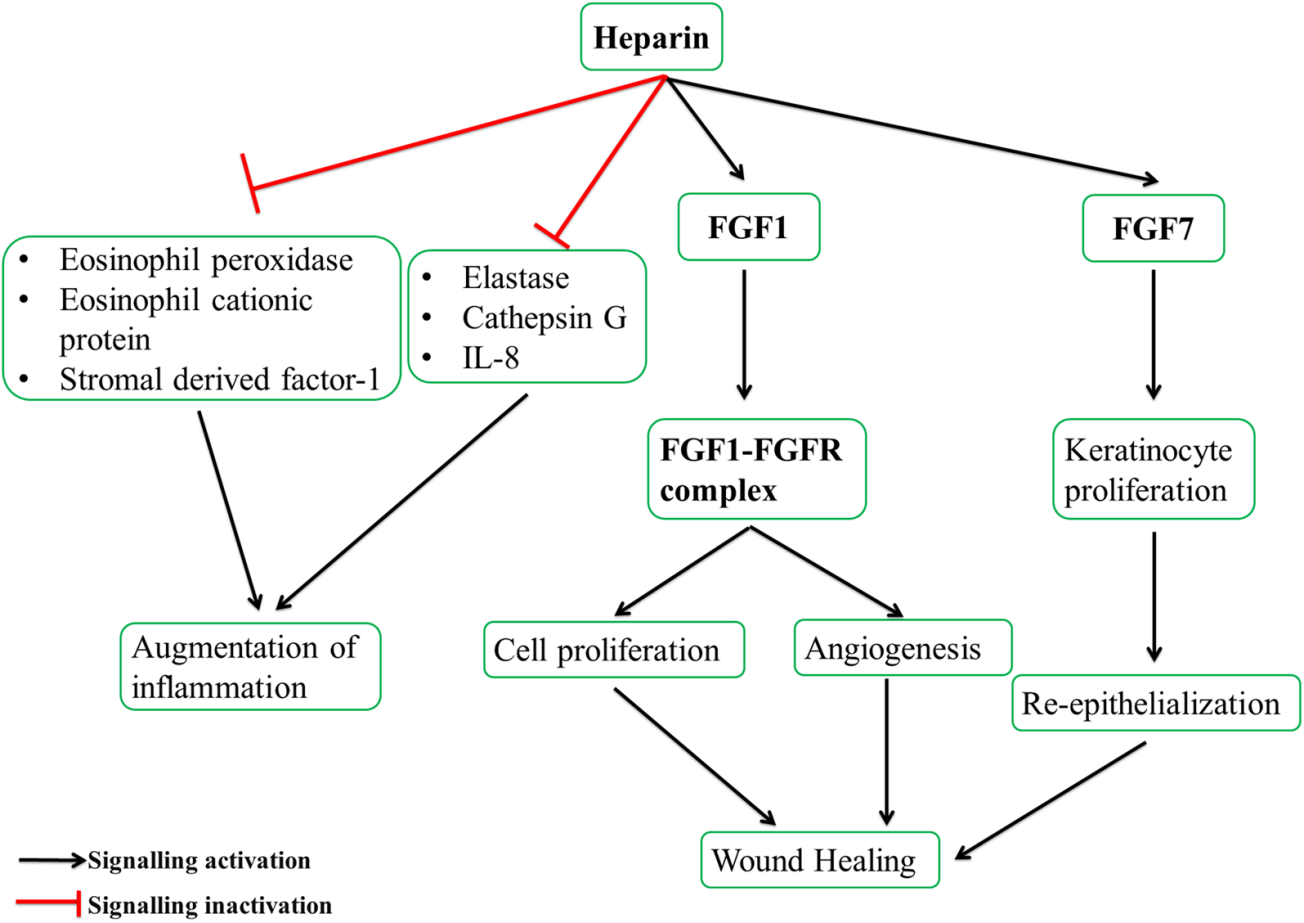
The figure demonstrates the signaling pathway followed by the heparin protein in wound healing. Heparin promotes the secretion of different fibroblast growth factors responsible for re-epithelialization, migration, and differentiation of growth-promoting cells

Heparin is a natural, branched, helical glycosaminoglycan (GAG) with high sulfation and anticoagulant properties. It is basically divided into two main types: unfractionated heparin and low molecular weight heparin. Among the naturally occurring GAGs, heparin is the most sulfated one and composed mainly of tri-sulfated disaccharides of 2-*O-*sulphated α-L-iduronic acid and N-,6-*O-*disulphated glucosamine repeating units (Fig. 2E). The molecular weight of natural heparin is 3000–30,000 Da, while that of unfractionated one is 12–16,000 Da [70]. It is effective in wound healing due to its ability to protect growth factors from proteolytic degradation, thus enhancing their bioactivity [71]. It promotes rapid and effective repair of endothelial cells, making it a useful agent in both in vitro and in vivo wound-healing applications [72]. Heparin has been employed in treating burn wounds and diabetic foot ulcers, where it has been shown to decrease wound recovery time and increase capillary circulation. The wound-healing capability of heparin-based formulations is due to their ability to enhance the secretion of various growth factors, such as FGF1 and FGF7. FGF1, after binding to its receptor FGF1-R, promotes cell proliferation and angiogenesis, while FGF-7 increases the proliferation of keratinocytes, which is essential for re-epithelialization [73]. Heparin also inhibits specific cytokines, including Elastase, Cathepsin G, and IL-8, as well as eosinophil peroxidase, eosinophil cationic protein, and stromal-derived factor-1, which are responsible for the augmentation of inflammation. Overall, heparin-based formulations have shown great potential for wound-healing applications due to their ability to promote cell proliferation, angiogenesis, and re-epithelialization, while inhibiting inflammation as shown in Fig. 6. Using heparin-based formulations in wound healing may lead to the development of novel therapies and biomaterials for improving wound-healing outcomes [71, 74].

### Fibroblast growth factor (FGF)

FGF plays a crucial role in wound healing and includes 22 polypeptides which are responsible for activating the FGFR1-4 (four receptor-type tyrosine kinases) and promotes healing [75]. The granulation tissue formation and angiogenesis are mediated by FGF2. FGF2 also promotes cell proliferation, differentiation, and migration in different tissues, including skin, bone, cartilage, and muscles [76]. Further, the migration of keratinocytes in wounds, both in vitro and on skin samples, is promoted by FGFR-1 and FGFR-2 [77]. FGF7 and FGF10 are responsible for stimulating the endothelial cells and inducing the expression of VEGF, both of which are required for re-epithelialization and angiogenesis in its later stages. Further, hair follicles are generated with the help of FGF9, and its overexpression increases hair generation up to three times. Keratinocyte growth factors (KGF) also fall under this category and promote reepithelialization by affecting the morphogenesis, proliferation, and migration of keratinocytes. KGF1 regulates the inflammatory phase, and its expression is increased by the PDGF and proinflammatory cytokines released from macrophages and leukocytes. KGF2 acts on epithelial cells and is secreted by fibroblasts. It promotes angiogenesis, hair follicle growth, wound closure, scar formation, fibroblast migration, granulation tissue formation, and so on [78, 79] (Fig. 2F, G).

### Vascular endothelial growth factor (VEGF)

Various cell types, including fibroblasts, keratinocytes, platelets, endothelial cells, macrophages, and neurocytes present in the wound site, express the growth factors of the VEGF family. VEGF plays a crucial role in initiating the scar formation and the early phases of angiogenesis after the tissue injury, and its expression is initiated by the release of hypoxia-inducible factor-1α in response to the blood capillary disruption and hypoxic conditions generated thereafter at the wound [79]. It also promotes vasculogenesis, re-epithelialization, collagen deposition, and enhanced vascular permeability, which allows the inflammatory cells to reach the wounded tissue and initiate proliferation and migration. It also assists in burn wound healing [80] (Fig. 2H).

### Epidermal growth factor (EGF)

It is a polypeptide chain having 53 amino acid residues and three intramolecular disulfide bonds. The migration and proliferation of fibroblast, endothelial cells, and keratinocytes toward the wound site is initiated by EGF. It also activates the EGF receptor (EGFR), which initiates the signaling pathway involved in promoting cell survival, proliferation, and migration without causing any harm to stem cell pluripotency. EGF is found to enhance the healing rate in diabetic foot ulcers, venous ulcers, and skin grafts by increasing epithelialization [81, 82] (Fig. 2I).

### Platelet-derived growth factor (PDGF)

PDGF is secreted in the form of five isoforms by fibroblasts, macrophages, smooth muscle cells, keratinocytes, and endothelial cells. PDGF proteins are present in monomeric forms, and to get themselves biologically active and bind to the PDGF receptors, they form homodimers or heterodimers. The differentiation and proliferation of fibroblasts to myofibroblasts is initiated by the PDGF. It plays a role in re-epithelialization, intraepithelial collagen deposition, inflammatory cell deposition, and stabilization of blood capillaries in granulation tissue. Further, it promotes the secretion of MMPs and thus is critical in the remodeling phase. The PDGF-BB (Becaplermin) is the only recombinant growth factor approved for chronic wound healing by FDA, and it acts as a profibrotic agent [79, 83] (Fig. 2J).

## Synthesis of protein-based nanoformulations

**Fig. 7:**
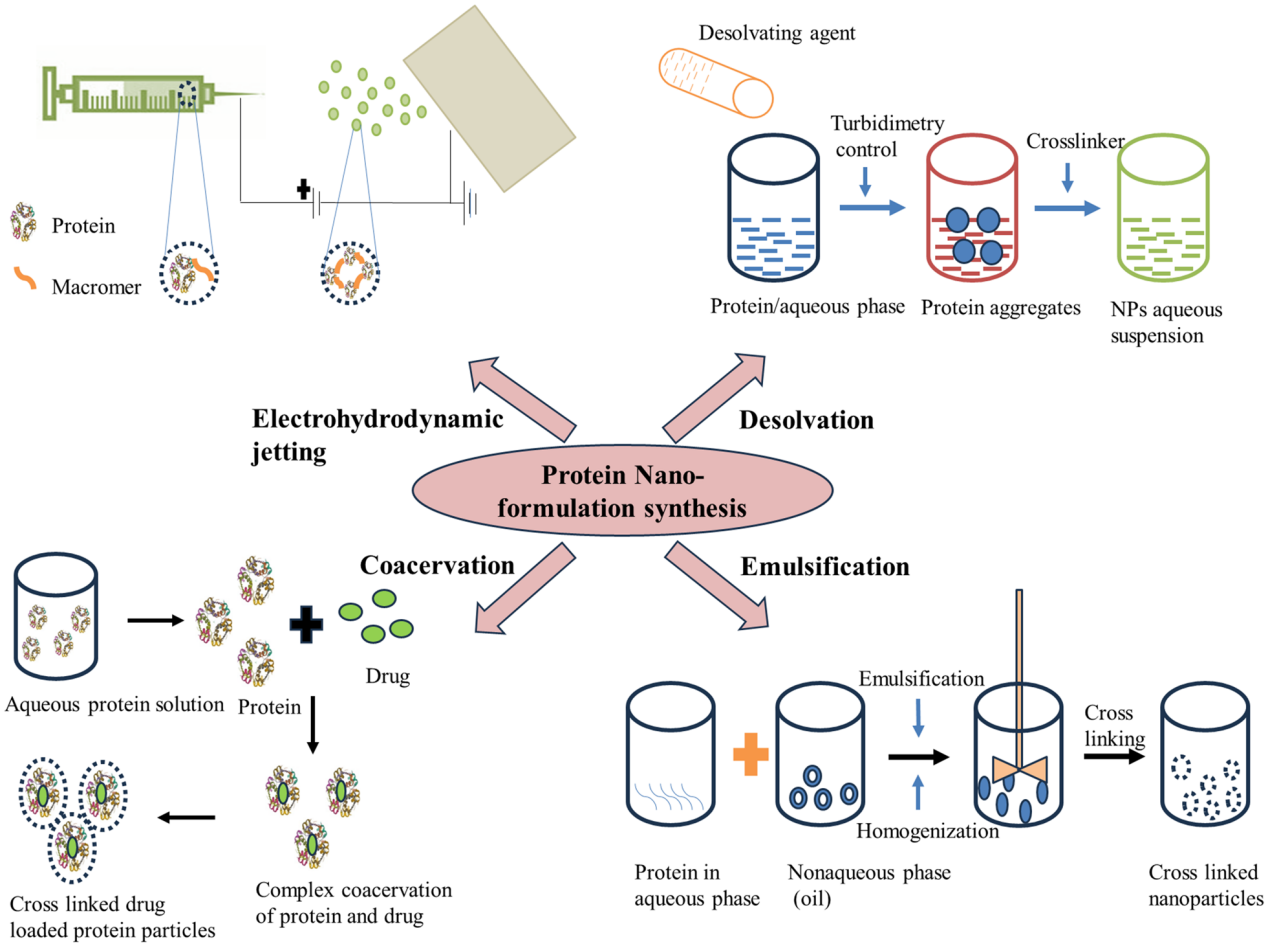
The figure provides information about the different techniques being followed for synthesizing the desired protein-functionalized nanoparticles and includes emulsification, desolvation, coacervation, and electrohydrojetting techniques

Nanoparticles are promising drug delivery agents for early diagnosis and treatment of various diseases. Different methods have been employed for their synthesis, including colloidal, sonochemical, thermal decomposition, microemulsion, and hydrothermal processes [84, 85]. However, these methods have limitations due to the toxicity of drugs on normal cells and tissues and the difficulty in loading hydrophobic agents [86, 87]. To overcome these limitations, protein-based nanoformulations have been developed. Two main synthesis pathways are crosslinking with derivative groups modified on the surface of protein molecules or crosslinking with native proteins' functional groups [88, 89]. Recent techniques involve the electrospray method and desolvation or coacervation process, which provide better control over the size and loading of nucleic acids and therapeutic drugs [90–93]. UV illumination was used to induce the self-assembly of protein nanoparticles, and solvent extraction or emulsion processes were found to have high encapsulation rates [94]. The heat denaturation process is equipped with targeting moieties but lacks a large particle size. Hence, various synthesis methods with modifications can be employed to efficiently and effectively synthesize protein-functionalized nanoparticles [90]. Here we are going to discuss some of the most widely employed synthesis methods used for protein-functionalized nanomaterials as shown in Fig. 7 and their advantages and limitations are given in Table 1 [95]. The synthesis procedure to be followed will be highly dependent on the specific application and the characteristic features of the resulting nanostructures.

**Table 1:** The table provides the brief information about the different synthesis techniques being followed along with some of the major advantages and disadvantages of each technique

Technique	Advantage	Disadvantage	References
Solvent extraction/emulsion process	The rate of encapsulation is very high Require basic laboratory equipment	Large particle size Poor drug loading Uncontrolled drug release	[96, 97]
Complex Coacervation	Performed under mild conditions High shell integrity Excellent drug release efficacy	Agglomeration chances are high Easily affected by temperature, pH, ionic strength, composition, and nature of the material	[98, 99]
Salt precipitation	A simple and robust technique	High chances of confirmation and bioactivity loss	[111]
Polyelectrolyte complexation	Encapsulation efficiency is high Maintains the drug stability	Affected by pH variations, temperature, ionic strength, polyelectrolyte concentration	[112]
Desolvation	Easy synthesis Low cost High yield	Protein denaturation Loss of biological activity Affected by the pH of the protein	[100, 101]
Heat denaturation	Targeting moieties can be attached Implemented on a large scale	Large particle size Not suggested for heat-sensitive compounds	[113]
UV illumination	Assist in the self-assembly of proteins	Chances of agglomeration	[94]
Layer-by-layer assembly	Multilayered structures Controlled size and surface charge Monodisperse particles Unlimited geometry of protein nanoparticles	Relatively low yield Computational calculations needed Affected by protein concentration	[104, 105, 114]
Electrohydrodynamic jetting	The secondary protein structure retained Able to trap hydrophobic and hydrophilic drugs Maintain the narrow dispersity of particles	Low yield Affected by the molecular weight of the protein	[102, 103]
Solvent extraction/emulsion process	The rate of encapsulation is very high Require basic laboratory equipment	Large particle size Poor drug loading Uncontrolled drug release	[96, 97]
Complex Coacervation	Performed under mild conditions High shell integrity Excellent drug release efficacy	Agglomeration chances are high Easily affected by temperature, pH, ionic strength, composition, and nature of the material	[98, 99]
Salt precipitation	A simple and robust technique	High chances of confirmation and bioactivity loss	[111]
Polyelectrolyte complexation	Encapsulation efficiency is high Maintains the drug stability	Affected by pH variations, temperature, ionic strength, polyelectrolyte concentration	[112]
Desolvation	Easy synthesis Low cost High yield	Protein denaturation Loss of biological activity Affected by the pH of the protein	[100, 101]
Heat denaturation	Targeting moieties can be attached Implemented on a large scale	Large particle size Not suggested for heat-sensitive compounds	[113]
UV illumination	Assist in the self-assembly of proteins	Chances of agglomeration	[94]
Layer-by-layer assembly	Multilayered structures Controlled size and surface charge Monodisperse particles Unlimited geometry of protein nanoparticles	Relatively low yield Computational calculations needed Affected by protein concentration	[104, 105, 114]
Electrohydrodynamic jetting	The secondary protein structure retained Able to trap hydrophobic and hydrophilic drugs Maintain the narrow dispersity of particles	Low yield Affected by the molecular weight of the protein	[102, 103]

### Emulsification technique

In 1972, the method was developed by Scheffel and co-workers for preparing the spheres of albumin protein. This technique involves two distinct phases, an aqueous phase is made up by dissolving the protein in distilled water, and an organic phase in which plant oils are used. The two phases are mixed mechanically by using a homogenizer in a large container until the oil–water or water–oil emulsion is obtained. This emulsion solution is then poured drop by drop into a preheated oil having a temperature of up to 120 °C. At this temperature, the water from the emulsion will evaporate, and the irreversible destruction of protein will begin, eventually leading to the synthesis of protein nanoparticles which will be suspended in an ice bath [96, 97].

### Complex coacervation

For the entrapment of DNA, a complex coacervation technique is usually followed. By adjusting the pH, the proteins can be made cationic or anionic due to their amphoteric nature. In this technique, the protein is taken in an aqueous solution, and during its pH adjustment duration, the particles having a positive charge come upward toward the surface. In this solution, a freshly prepared solution of DNA mixed with salt is added. When the DNA and protein interact together, the complex coacervation occurs, followed by the addition of crosslinkers like EDC (1-ethyl-3-(3-dimethyl aminopropyl)carbodiimide) to get the crosslinked protein particles loaded with the DNA. This entrapment of DNA into the protein matrix is completed in the last stage of synthesis. Cationized protein can also be used for making complexes with DNA [98, 99].

### Desolvation

Desolvation, also known as the coacervation technique, was developed in 1978 by Martyr and his coworkers. In this technique, protein is taken in an aqueous solution into which a desolvating agent like natural salt or alcohol is added. Adding a desolvation agent starts slow structural changes in the protein after addition. After a specific time interval, the formation of protein clumps begins, followed by the crosslinking between clumps to yield protein nanoparticles which are separated from the solution by gradually increasing the turbidity of the solution [100, 101].

### Electrospray technique

The electrospray technique is a newly developed one mainly used for elastin and gliadin-based protein nanoparticles. In this technique, a very high voltage is applied to the protein solution, which is supplied through the emitter. This emitter is responsible for emitting a liquid jet stream via the nozzle, which is crucial for forming aerosolized size liquid droplets. These droplets consist of drugs along with nucleic acid. Through this method, the monodispersity of synthesized nanoparticles is retained [102, 103].

### Other techniques

In addition to the methods mentioned earlier, other techniques have been developed to synthesize protein-based nanoformulations. One such technique is the layer-by-layer (LbL) assembly method, which involves the sequential deposition of oppositely charged polyelectrolytes onto a charged surface, such as a protein or a nanoparticle. This method is highly versatile and can be used to create multilayered nanostructures with controlled size and surface charge, allowing for precise control over the release of drugs or other therapeutic agents [104, 105]. Another method that has gained attention in recent years is using genetically engineered proteins to synthesize protein-based nanoformulations. By engineering the amino acid sequence of proteins, it is possible to introduce specific functional groups that can be used for crosslinking or chemical modification, allowing for the precise control of the size and shape of the resulting nanostructures [106, 107]. Furthermore, there is a growing interest in using biocompatible and biodegradable polymers, such as chitosan, alginate, and poly(lactic-co-glycolic acid) (PLGA), for the synthesis of protein-based nanoformulations. These polymers offer several advantages over traditional protein-based formulations, including improved stability, enhanced biocompatibility, and controlled release properties [108–110].

## Kinetic and thermodynamic study of protein-based nanoformulations

Thermodynamic and kinetic studies are crucial in thoroughly understanding the interactions between proteins and external agents such as drugs, metal ions, polymers. Thermodynamics critically affect the different parameters including stability, adsorption, nucleation, and interaction between proteins and the surface of nanoparticles and helps in the determination of optimal conditions for the development of protein-functionalized nanomaterials having desired characteristic features and applications. The stability of protein is very crucial for its bioactivity at the targeted site, and with the help of thermodynamics, one can study the structural alterations in proteins which can be due to various environmental or man-made parameters ultimately causing its misfolding or deactivation and eventually resulting in losing the stability. The surface adsorption of proteins on the molecular surface depends upon certain factors such as size, shape, composition, and the extent of binding interactions both at the surface and within the molecules. The formulations show wide variations in behavior when different proteins are used, which further depends upon the bulk solution constituents, the ratio of sizes between the proteins used, and protein–surface interactions. Additionally, the potential of different proteins to alter their structure after getting adsorbed greatly affects the kinetics of adsorption. Different adsorption patterns can be obtained based on the internal rates of structural modifications when compared with the protein diffusion, which can also be determined using intermolecular interactions.

Moreover, there is a strong relation between various relaxation times with the kinetics of adsorption and depends upon the morphology of particles [115]. A convex isotherm is formed when there is an association between protein and immobilized ligands. In contrast, when a protein molecule is adsorbed on another protein molecule, a sigmoid isotherm is formed, which is concave at low concentrations while convex at higher concentrations. For instance, insulin has concave isotherms at low concentrations [116]. One needs to study different parameters to understand the effect of all these factors. The thermodynamic parameters consist of Gibbs free energy (Δ*G*), enthalpy (*H*), and entropy (*S*), which can be calculated by using the following set of equations for all kinds of protein-based formulations.

1 $$G = - RT\;{\text{ln}}\;K$$

2 $${\text{ln}}\;K = - H/RT + S/T$$

3 $$G = H{-}TS$$

where *T* is the temperature, and *R* represents the universal gas constant.

The other factors include *K*_a_ (association constant) and *K*_d_ (dissociation constant). Different protein-based formulations have different values for these parameters, which are briefly explained using some examples. The adsorption isotherms were plotted to determine the relationship between molecules that get absorbed on the surface and those remaining in solution to monitor the binding capacity between the insulin protein and different magnetic nanoparticles (MMIP and MNIP). The slope of the adsorption curve increases sharply when a low initial concentration of the sample is used, while at higher concentrations, the slope is almost constant. Additionally, the ∆*G* values (in kJ/mol) for MMIP and MNIP are − 39.2 and − 36, the *H* values (in kJ/mol) are − 39.3 and − 36, while the ∆S values (in J/mol K) are − 0.26 and − 0.27. The negative values of ∆*G* indicate that insulin adsorption is a spontaneous process.

Conversely, the negative values of ∆*H* and ∆*S* indicate that electrostatic forces, hydrogen bonds, and van der Waals forces are involved in insulin adsorption. Similarly, equilibrium results were evaluated using Langmuir and Freundlich isotherms to clarify the binding mechanisms of proteins with external agents. The adsorption kinetic model was used to study the kinetic mechanism of insulin adsorption, and it was observed that adsorption occurs via a pseudo-first-order mechanism during the initial 8 h due to the presence of empty binding sites and lower concentrations of molecules, while after 8 h, adsorption occurs via a pseudo-second-order reaction [117]. Similarly, novel nanoinsulin formulations were developed using silver nanoparticles. Their ∆*G* values were found to be − 6.72, − 6.98, and − 7.48 kcal/mol at 27 °C, 32 °C, and 37 °C, respectively, indicating the favorability of the forward reaction with the highest affinity at physiological conditions. Similarly, the ∆H value is 16.08 kcal/mol, indicating the endothermic nature of the reaction between insulin and AgNPs. Furthermore, it was found that the reaction followed a first-order kinetic model based on the fluorescence quenching of insulin [49]. In the case of regenerated silk fibroin, crystallization is exothermic and is accompanied by entropy reduction when the temperature is kept constant. As the draw ratio increases, thermal stability and crystallinity are observed [118]. For the adsorption of heparin molecules on different molecularly imprinted polymers, the ∆*G* values were found to be − 5.95, − 4.70, and − 2.73 kJ/mol at 299.15 K, 309.15 K, and 324.15 K, respectively, confirming the feasibility and spontaneity of the adsorption process. Moreover, the values of ∆*H* are negative, indicating electrostatic interactions between molecules, while the ∆*S* values are also negative, implying a decrease in entropy due to decreased randomness at the solution-solid binding point [119]. Further, the energy expenditure during the different wound-healing phases varies greatly by 50% and 20% in proliferative and remodeling phases. Also, the thermodynamics greatly influence the inflammatory phase by affecting the key features including redness, swelling, and heat production during inflammation of which swelling and redness are influenced by osmotic pressure and fluid movement. The fluid movement raises by 100 times in order to meet the wound requirements by supplying nutrients and blood to the tissue site. Gibb’s free energy plays an important role in wound healing as it affects the cell migration, collagen deposition, and angiogenesis. The healing wounds have a positive ∆G indicating the progress through phases of wound healing. Chronic wounds have negative ∆G which indicates that the wound-healing process is stuck in inflammatory phase and cannot be proceeded. In this way, kinetic and thermodynamic factors play a crucial role in understanding the stability of nanoparticles and the feasibility of synthesizing protein-based nanoformulations for further applications.

## Protein-based nanoformulations in normal and diabetic wound healing

### Insulin-based formulations

**Fig. 8:**
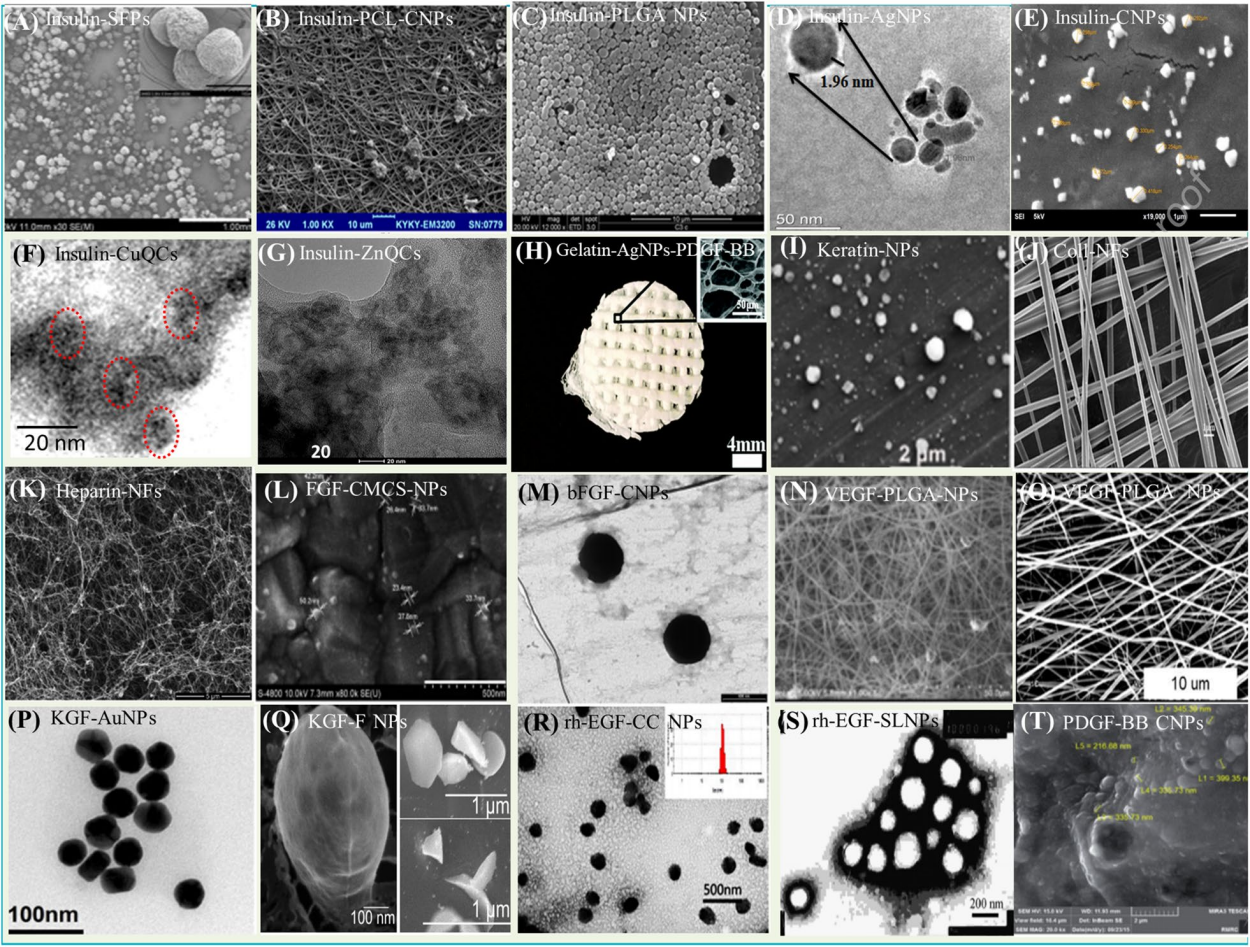
SEM**/**TEM images of the different protein-loaded nanoformulations **A** Insulin-silk fibroin nanoparticles, **B** Insulin-PCL-Chitosan nanoparticles, **C** Insulin-PLGA nanoparticles, **D** Insulin-Ag nanoparticles, **E** Insulin-Chitosan nanoparticles, **F** Insulin-Cu Quantum clusters, **G** Insulin-Zinc quantum clusters, **H** Gelatin-AgNPs-PDGF-BB, **I** Keratin nanoparticles, **J** Collagen nanofibers, **K** Heparin nanofibers, **L** Fibroblast growth factor-CMCS nanoparticles, **M** bFGF-loaded chitosan nanoparticles, **N** VEGF-PLGA nanoparticles, **O** Vascular endothelial growth factor-loaded PLGA nanoparticles, **P** Keratin Growth factor-Au nanoparticles, **Q** KGF-loaded fibrin nanoparticles, **R** rh-EGF-loaded carboxymethyl chitosan nanoparticles, **S** rh-EGF-loaded solid lipid nanoparticles, **T** PDGF-BB nanoparticles [Adapted with permission from ACS, RSC, Elsevier, Taylor and Francis, Springer Nature, MDPI], all rights reserved

Insulin has anti-inflammatory, antidiabetic, and wound-healing properties by activating the cytokines, thus reducing inflammation. Insulin upregulates the NF-kβ^P50/P50^ by suppressing the expression of NF-kβ^P50/P65^ and TNF-α. P65 suppression downregulates IL-12, IL-1β, TNF-α, and IL-6 cytokines at the wound site [120–122]**.** Inflammatory cytokines inhibition shifts the equilibrium toward anti-inflammatory cytokines expression, like VEGF, IL-4, IL-10, etc., which further induces the proliferation of the cells [123, 124]. Numerous approaches for developing nanoformulations of insulin have been carried out. In 2017, Li and co-workers developed silk fibroin-based microparticles (insulin-SFPs) encapsulating insulin; the particles provide biostability to the insulin and help in its sustained release. The particles showed significant collagen deposition, and vascularization stimulated the migration of keratinocytes and endothelial cells and promoted wound healing compared to the free insulin [125, 126] (Fig. 8A). In 2018, Ehterami et al. followed ion gelation method for preparing insulin-loaded chitosan particles (insulin-CPs) followed by their coating on poly ε-caprolactone (PCL) (insulin-PCL-CNPs); the particles showed a reduction in inflammatory cytokines infiltration and 96.9 ± 1.11% wound healing in 14 days [127] (Fig. 8B).

In 2018, DH Abdelkader et al. developed insulin-loaded polyvinyl alcohol borate nanoparticles (insulin-PLGA CNPs), significantly enhancing wound healing. With PLGANP-insulin, 29.15% more wound recovery occurs on the 10th day of the treatment. The insulin embedded in the particles is released by the diffusion method. The difference in wound closure up to 16 days in diabetic rat models treated with control, free insulin, and insulin-loaded PLGA particles showed faster wound healing and increased exudates formation and angiogenesis with insulin PLGA insulin particles [128] (Fig. 8C). In 2019, Kaur P et al. prepared insulin protein-coated silver nanoparticles (insulin-AgNPs) to deliver insulin at the place of injury (Fig. 8D). Along with anti-bacterial effect, AgNPs also have significant role in treating wounds and exhibit anti-inflammatory action. The inflammatory cytokine transition and wound-healing properties of AgNPs improved by encapsulating them with insulin. The wound-healing effect of IAgNPs was determined in vitro and in vivo in diabetic and non-diabetic conditions. Both free insulin and IAgNPs treatment showed a faster recovery of the wound. 20% and 12%, more rapid wound recovery in diabetic and normoglycemic rats, respectively, on the 5th day of IAgNPs treatment than free insulin 4.67% (diabetic model) and 7.27% (non-diabetic model). After 11 days of IAgNPs treatment, 73.33%, 60.0%, and free insulin, 40% and 33.33%, were observed in diabetic and normoglycemic models, respectively, compared to the respective controls. In addition to the wound contraction assay, the histological expression of the pro- and anti-inflammatory cytokines was also examined. Histological evaluations of rat models on the 5th and 11th days showed significant collagen deposition along with reepithelialization. A decrease in the leukocyte infiltration was observed with IAgNPs and insulin with respect to additional solutions. On the 5th day of treatment, IAgNPs, the pro-inflammatory cytokines expression level decreases by 50% in both diabetic and non-diabetic groups, which is higher than free insulin. On the 11th day, IL-6 and TNF-α decreased by 45% in diabetic and normoglycemic sets. The IL-10 anti-inflammatory cytokines expression level after 5 days of treatment with IAgNPs increases by 70% and 50% in non-diabetic and diabetic rat animals. On the 11th day, wound healing accelerated by 65% and 50% in non-diabetic and diabetic groups [49]. Ribeiro et al. [130] used chitosan to synthesize insulin-loaded nanoparticles (insulin-CNPs) and examined the effect on wound healing. Histological evaluation showed angiogenesis, reduction in inflammation like monocytes, macrophages, neutrophils, keratinocytes, endothelial cell proliferation, collagen deposition, and wound maturation after treatment compared to free insulin [129] (Fig. 8E). The particles of Poly (lactide-co-glycolide) (insulin-PLGA NPs) loaded with insulin were prepared by Lee and Blaber [131] by evaporating the solvent using the double-emulsion technique. The prepared particles are biodegradable and have viscoelastic properties—the insulin embedded in the particles is released by diffusion. The difference in wound closure in control, free insulin, and insulin-loaded PLGA particles was up to 16 days. With insulin PLGA particles, the healing rate is faster and increases the exudate formation and angiogenesis formation [130].

The nanoclusters (NCs) of insulin with metals have bioimaging properties and wound-healing activity. These NCs comprise several to a hundred metal atoms with an outer layer of protein to protect them from aggregation. Kaur et al. [14] prepared insulin copper quantum clusters (Insulin-CuQCs) that exhibited wound healing and bioimaging activity. Similar to zinc, copper also promotes cell growth and division. At a 5% concentration of ICuQCs in 24h, there was an almost double increase in HeLa cell growth compared to the control (insulin + Cu salt, insulin, and blank), indicating the ICuQCs' cell growth-promoting effect [14] (Fig. 8F). Kaur et al. [15] prepared insulin zinc quantum clusters (Insulin-ZnQCs) and tested their in vitro wound-healing efficiency and bioimaging activity using HEKa (normal human epidermal keratinocyte). The cells treated with IZnQCs at concentrations of 1.5, 7.5, 30, and 60 µM showed 39.49 ± 1.29%, 42.03 ± 3.04%, 45.25 ± 2.14%, and 52.88 ± 0.83% cell migration, respectively, after 6 h compared to control. After 12 h of incubation, cell migration of 27.58 ± 3.72%, 34.08 ± 1.57%, 36.32 ± 1.63%, and 46.86 ± 1.46% occurred with 1.5, 7.5, 30, and 60 µM IZnQCs treatment compared to control. After 24 h, the % of cell migration was 43.02 ± 1.62%, 46.51 ± 3.38%, 58.60 ± 0.72%, and 67.81 ± 0.83% after 1.5, 7.5, 30, and 60 µM IZnQCs concentrations treatment, respectively, in comparison with control. An increase in migration was observed with an increase in time, and at 24h, maximum wound recovery was observed [15] (Fig. 8G). Similarly, insulin-nickel quantum clusters (INiQCs) were prepared by Sharda et al. to test wound-healing efficiency in vitro, bioimaging, and detecting lead in silico and in vitro. They effectively healed wounds at different concentrations of 1.5, 7.5, 30, and 60 µM after different time intervals of 6, 12, and 24 h, respectively, and showed bioimaging effects at varying concentrations [16]**.** Also, insulin-cobalt core–shell nanoparticles were synthesized for the treatment of wounds and are found to be very effective against both normal and diabetic wound healing. The effect of particle concentration was studied, and it was found that with increasing concentrations of 1.5, 7.5, 30, and 60 µM, there is an increase in the rate of wound healing with time [131]. A hydrogel scaffold was developed using morin incorporated into polysaccharide protein, which decreased the re-epithelialization rate and increased the wound contraction rate by enhancing collagen deposition in diabetic rats [132]. Wan et al. [133] prepared Gelatine cryogel loaded on the surface of silver nanoparticles (Gelatin-AgNPs-PDGF-BB) at the PDGF-BB bottom layer, which exhibited better wound healing, re-epithelialization, angiogenesis, deposition of collagen, and granulation tissue formation in diabetic wounds (Fig. 8H).

### Silk fibroin-based formulations

Curcumin-loaded silk fibroin conjugated with polycaprolactone or polyvinyl alcohol was used to make a nanofibrous mat for healing diabetic wounds faster in both in vitro and in vivo models [134]. Maity et al. [135] synthesized antioxidant silk fibroin composite hydrogel to heal diabetic wounds rapidly. They were biocompatible, stimulated fibroblast cell migration, and controlled oxidative stress in vitro. Silk fibroin and silk sericin-based formulations were developed in combination with aloe vera gel for wound healing in diabetic mice, and it was found that the wounds healed within 13 days of applying the formulation, with a wound contraction of 98.33 ± 0.80% [136]. Hydrogel films were created by combining boric acid-impregnated silk fibroin/gelatin and hyaluronic acid, resulting in an improved tissue healing process and increased strength [137]. Silk fibroin interlinked with glycyrrhizic acid and silver hydrogels was also prepared for the effective treatment of wounds having bacterial infection [138]. Additionally, a multitasked aerosolized nanopowder formula made from silk fibroin exhibited antioxidant, anti-bacterial, and enhanced cell proliferation effects, providing a promising approach to wound healing [139]. Wu et al. [140] found that adipose-derived stem cell-seeded silk fibroin-chitosan films improved wound healing in a diabetic rat model. Sen et al. [141] synthesized silk fibroin-immobilized polyurethane conjugated with epidermal growth factor, which enhanced the rate of burn wound healing of full-thickness burn and reduced pro-inflammatory cytokines IL-6,8,10 levels in diabetic rats. Silk fibroin-integrated biliverdin-based bioinspired green hydrogel-stimulated angiogenesis and wound healing in a full-thickness rat model by exhibiting anti-inflammatory effects in vitro and in vivo [142]. Silk fibroin-hyaluronic acid-based composite scaffolds were developed for monitoring the cellular growth at wound sites and were found to enhance the scarless reconstruction of skin in nude mice with skin tissue defects [143]. Silk fibroin and poly(lactide-co-glycolic acid) nanofiber were loaded with zinc oxide nanoparticles for their successful delivery at the wound site and to promote reepithelialization, collagen deposition, granulation tissue formation, and angiogenesis [144].

### Keratin-based formulations

Veerasubramanian et al. [145] developed konjac-glucomannan-keratin hydrogel wound dressings having oat extract, which significantly enhanced the wound treating ability in diabetic conditions by promoting the synthesis and deposition of collagen in the wounded area. In another study, recombinant keratin particles (Keratin-NPs) were prepared by Gao et al. [146] to improve wound healing, vascularization, epithelization, and collagen deposition (Fig. 8I). Chicken feather proteins were utilized by Kumaran et al. [147] to synthesize keratin hydrogels for treating dermal injuries. Furthermore, keratin hydrogels loaded with ciprofloxacin were developed to promote the healing of full-thickness excision wounds and prevent infection caused by *Pseudomonas aeruginosa* bacteria [148]. Human platelet lysate-loaded keratin hydrogels were also synthesized to exhibit sustained release of pro-regenerative growth factors essential for healing and enhancing cell proliferation without toxicity in vitro models [149]. Robert et al. [150] utilized keratin-based wound dressing to treat a patient suffering from recessive dystrophic epidermolysis bullosa in 2012. Electro-spun fibers, loaded with keratin and hyaluronic acid, were also found to be potent wound-healing dressings due to their ability to increase cell viability and proliferation [151]. Keraderm is a matrix dressing obtained from keratin powder by freeze-drying which is utilized for healing exuding venous ulcers and healing the complete wound in 30 weeks. Similarly, kerafoam, an absorbent polyurethane foam dressing having a keratin film lamination, was developed for highly exuding venous ulcers, which get completely healed in 16 weeks. To treat partial-thickness wounds, an absorbable matrix called keramatrix was developed, which is found to enhance the epithelialization process of wounds [152].

### Collagen-based formulations

Other extracellular proteins, such as amnion and collagen-based composite hydrogels, were developed to treat cutaneous burn wounds by enhancing the re-epithelialization abilities [153]. Curcumin-loaded fish scale collagen-hydroxypropyl methyl cellulose nanogel promoted healing with better percent contraction of the injury and prolonged drug release [154]. Chitosan/collagen scaffolds loaded with Norfloxacin were also developed to enhance skin reconstruction and improve adhesivity and mechanical strength for healing [155]. Sun et al. [156] prepared collagen nanofibers (Coll-NFs) in 2018, which were found to decrease inflammation, promote faster recovery of wounds, and increase angiogenesis (Fig. 8J). A matrix of Collagen-laminin having resveratrol loaded with hyaluronic acid-DPPC microparticles was used in treating the injury in diabetic rats, and a controlled drug release was achieved along with its antioxidant activity [157]. Collagen-chitosan scaffolds loaded with pioglitazone were synthesized, which were biocompatible, promoted cell growth, and exhibited enhanced wound contraction in diabetic wounds [158]. In yet another formulation, collagen dressing was loaded with neurotensin, which facilitated the controlled release of drugs at the site of injury, enhanced re-epithelialization, and decreased the secretion of inflammatory cytokines in diabetic foot ulcers [159].

### Heparin-based formulations

Heparin microislands were developed by Pruett et al. [160] in microporous scaffolds, which promoted diabetic wound-healing abilities by epidermal regeneration and re-vascularization. The heparin-poloxamer hydrogel was also developed by Xu et al. [161] using polylysine to heal endometrial injury by controlling KGF release and enhancing adhesiveness. Double-emulsion nanoparticles were developed using sulfated alginate and polycaprolactone to improve the delivery of heparin-binding growth factors that promote healing due to connective tissue growth factor secretion [162]. Additionally, pro-angiogenic nanofibrous membranes based on chitosan and heparin were developed using an efficient and new electrospinning method for wound-healing applications, which enhanced tissue angiogenesis [163]. Senturk et al. [164] prepared heparin memetic particles (heparin-NFs) in 2016, which were found to enhance re-epithelialization, promote a fast transition of pro-inflammatory to anti-inflammatory cytokines, improve angiogenesis, and lead to high VEGF levels and wound recovery (Fig. 8K). Analyses of re-epithelialization, granulation tissue formation, blood vessel density, VEGF, and inflammatory response measurements quantified wound recovery [160]. The synthesis of heparin-based hydrogel incorporating Cu5.40 nanozymes was done for the inhibition of inflammation by decreasing the pro-inflammatory cytokines and ROS scavenging and eventually leading to wound healing [165]. Lu et al. [166] developed a thermoresponsive hydrogel involving heparin protein with *Lactococcus* incorporated in it, which is helpful in wound healing by promoting angiogenesis, reducing the inflammatory microenvironment across the wound area, and decreasing the risk of systemic toxicities by preventing the entry of bacteria at the infection site.

P311 peptide-based micelles were assembled with ROS-responsive polymer for the transformation of an oxidative wound environment to a regenerative environment for wound healing by promoting collagen deposition, re-epithelialization, cell migration, and granular tissue synthesis [167]. Recently Ge et al. developed a novel nanoarmor that mimics the earth’s defense mechanism for the transport of IL-4 by protecting its biological activity and enhancing circulation throughout the blood. The synthesized copolymer consists of two layers: outer polyethylene glycol layer and intermediate rosamirinic acid layer for protecting the innermost IL-4, which is helpful in ROS scavenging, decrease in the secretion of inflammatory cytokines, and M1 to M2 transition crucial for healing [168]. The different nanoformulations obtained by using different proteins along with their potential outcome are given in Table 2.

**Table 2:** The table provides the information about the different protein-functionalized nanomaterials along with the protein used, their key outcomes, and the models on which they were tested to give a detailed knowledge of different formulations

Protein	Formulation	Tested on	Key outcome	References
Insulin	Insulin-encapsulated silk fibroin microparticles	HaCaT Sprague Dawley rats	Sustained release of insulin Enhanced collagen deposition and vascularization The accelerated wound closure rate	[126]
	PCL/COLL loaded with insulin chitosan nanoparticles	L929 Male Wistar rats	Enhanced blood compatibility Protection against bacterial infection Enhanced cell adhesion, growth, and migration	[126]
	Insulin-loaded Poly(vinyl alcohol)-borate hydrogels	Male Sprague Dawley rats (diabetic)	Topical insulin delivery Granulation tissue formation rate and angiogenesis accelerated The localized rise in collagen deposition	[130]
	Insulin-loaded chitosan nanoparticles	Female Wistar rats (diabetic)	Stimulated vasodilation, proliferation, leukocyte chemotaxis Accelerated epithelialization and collagen deposition	[127]
	Insulin-loaded core–shell cobalt nanoparticles	HEKa	Enhanced cell migration and wound closure rate Targeted drug delivery Highly potent for bioimaging	[131]
	Zinc insulin quantum clusters	HEKa	Enhanced cell migration and wound closure rate Targeted drug delivery Highly potent for bioimaging	[15]
	Insulin-loaded silver nanoparticles	HEKa Male Wistar rats	Regulation of balance between pro and anti-inflammatory cytokines Enhanced cell migration and wound closure rate Targeted drug delivery	[14]
	Insulin nickel quantum clusters	HEK-293	Enhanced cell migration and wound closure rate Targeted drug delivery Highly potent for bioimaging In vitro and in silico lead detection	[16]
Silk Fibroin	Curcumin-loaded polycaprolactone/polyvinyl alcohol-based silk fibroin-based electrospun nanofibrous mat	Albino female mice	Antioxidant and anti-inflammatory activity Restore the skin structure and histology of tissue at a faster pace Faster wound healing	[134]
	Melanin and berberine-amalgamated silk fibroin hydrogel	NIH3T3 Wistar rat model (diabetic)	Enhanced biocompatibility Prevent oxidative stress Enhanced migration of fibroblasts, re-epithelialization, and wound closure	[135]
	*Aloe vera* gel-loaded silk fibroin formulations	Albino mice (diabetic)	Enhanced growth of collagen fibers and blood vessels Much decreased inflammation	[136]
	Silk fibroin/gelatin/hyaluronic acid-based films impregnated with boric acid	L929	Increased cell migration activity in vitro Promoted wound healing	[137]
	Silk fibroin crosslinked glycyrrhizic acid and silver hydrogel	*Staphylococcus aureus* infected wound in mice Chorioallantoic membranes	Enhanced biocompatibility and mechanical properties Antibacterial and anti-inflammatory effects Promote tissue regeneration	[138]
	Aerosolized nanopowder having neomycin and *Avicenna marina* extract loaded in silk fibroin	CRL2522	Potent increase in cell viability Enhanced antioxidant activity Excellent antibacterial activity	[139]
	Adipose-derived stem cell-seeded silk fibroin chitosan film	Male Sprague Dawley rats (diabetic)	Accelerated cell growth Differentiation of adipocyte and osteocyte enhanced Enhanced granulation tissue formation, capillary formation, re-epithelialization	[140]
	Epidermal growth factor-conjugated silk fibroin-immobilized polyurethane	NIH3T3 Rat model (normal and diabetic)	Enhanced granulation, collagen deposition, re-epithelialization Restoration of proinflammatory cytokines IL-6,8,10 No sign of infection at the wound site	[141]
	Biliverdin silk fibroin hydrogel	GL261 Male BALB/c nude mice	Enhanced anti-inflammatory effect Stimulated angiogenesis Full-thickness wound healing	[142]
	Hyaluronic acid containing silk fibroin scaffold	HUVEC Sprague Dawley rats	Enhanced cell adhesion, proliferation, and differentiation Regulation of collagen arrangement Inhibited scar formation	[143]
	Zinc oxide-loaded PLGA/silk fibroin electrospun membranes	L929 Male Sprague Dawley rats	Enhanced re-epithelialization, collagen deposition, granulation tissue formation, and angiogenesis Antioxidant and antibacterial activity	[144]
Keratin	*Avena sativa* extract-loaded konjac glucomannan-keratin hydrogel scaffold	NIH/3T3 Male Wistar rats	Exhibit desirable swelling, biocompatibility, antibacterial, and antioxidant activity Enhanced wound contraction and collagen synthesis Increased epidermis layer and blood vessel synthesis	[145]
	Chlorhexidine-loaded polysulfobetaine/keratin hydrogel	L929 Sprague Dawley rats	Reduced inflammation Enhanced collagen deposition Antioxidant and antibacterial	[169]
	Recombinant keratin nanoparticles	HaCaT Male Sprague Dawley rats	Enhanced cell migration and proliferation Improved epithelialization, vascularization, remodeling, and collagen deposition No systemic toxicity in vivo	[146]
	Chicken feather keratin hydrogel	Skin cells	Enhanced migration of keratinocytes from the boundary to the inner wound site	[147]
	Ciprofloxacin-loaded keratin hydrogel	Female Yorkshire pigs *P. aeruginosa*	Decrease in the amount of *P. aeruginosa* by 99% Collagen-rich granulation tissue and fibroblasts at the wound site Enhanced re-epithelialization and wound contraction	[148]
	Keratin hydrogel loaded with human platelet lysate	HDF	Sustained release of pro-regenerative growth factors Promoted and supported cell proliferation without causing toxicity for upto three days	[149]
	Keratin-based dressing (keragel T)	Patient with recessive dystrophic epidermolysis bullosa	Enhanced keratinocyte proliferation and migration Accelerated epithelialization, epidermal migration, and keratinocyte activation	[150]
	Nanocomposites based on polycaprolactone/polyethylene oxide loaded with hyaluronic acid and keratinin	L929	Non-toxic nature Enhanced cell proliferation and viability	[151]
Collagen	Curcumin-loaded fish scale collagen hydroxypropyl methylcellulose nanogel	Male albino Wistar rats	High stability, wound contraction ability, and safe for dermatological application	[154]
	Collagen/chitosan-based porous scaffold	HDF Male Wistar rats	Enhanced fibroblast migration, and proliferation Increased collagen deposition and synthesis of Collagen IV angiogenesis	[170]
	Mupirocin-loaded chitosan microspheres embedded in collagen scaffold	Unisex Wistar rats	Collagen deposition Fibroblast proliferation No inflammation	[171]
	Norfloxacin-loaded collagen/chitosan scaffold	Albino rats	High water uptake and retention ability Enhanced bioadhesive strength and slow biodegradation 100% drug release ability Accelerated tissue regeneration Intact epidermal and dermal structure	[155]
	Collagen scaffold	STZ-induced diabetic rat	Accelerated migration of fibroblasts and keratinocytes Enhanced angiogenetic activity Decreased inflammation	[156]
	Collagen-laminin dermal matrix impregnated with resveratrol-loaded hyaluronic acid-DPPC microparticles	Male Wistar albino rats	Excellent antioxidant activity eventually promotes wound healing Enhanced cell proliferation of fibroblasts, keratinocytes, and endothelial cells Maintain matrix integrity by acting against collagenase	[157]
	Pioglitazone-loaded collagen/chitosan scaffold	3T3-L1 Male wistar albino rats	Optimum porosity, low matrix degradation, sustained drug release Significant decrease in matrix metalloproteinases-9 Better growth of fibroblast Potent anti-inflammatory effect	[158]
	Neurotensin-loaded collagen matrix	HaCaT Raw 264.7 Male C57BL/6 Mice	Reduced inflammatory cytokine expression Decreased inflammatory filtrate and metalloproteinases Increased fibroblast migration Enhanced deposition of collagen, collagen type III, and alpha 1	[159]
Heparin	Heparin microislands	Mice HDF HDMVEC	Epidermal regeneration and revascularization in a diabetic model Downstream cell migration in vitro	[160]
	Heparin/Prussian blue-loaded nanofibers	NIH3T3 C57/BL mice	Decreases intracellular ROS level Prevent cells from ROS-mediated apoptosis Accelerate the inflammatory response and wound healing	[172]
	Heparin-based sericin hydrogel-encapsulated fibroblast growth factor	BALB/c mice HDF	Enhanced adhesiveness Excellent biodegradability Increased collagen deposition, vascularzation, and re-epithelialization	[173]
	Heparin poloxamer	ECC Female Sprague Dawley rats	Enhanced proliferation and angiogenesis of endometrial epithelial cells Inhibit cellular apoptosis in the endometrium	[161]
	Heparin mimetic alginate sulfate/polycaprolactone nanoparticles	HaCaT	Controlled drug delivery Protection of drug from degradation Enhanced cellular migration, proliferation, and matrix deposition	[162]
	Heparin-loaded chitosan nanofibers	chorionic allantoic membrane	Stimulated angiogenesis	[163]
	Heparin mimetic peptide nanofibers	Male Sprague Dawley rats (Diabetic)	Promote angiogenesis, re-epithelialization, and inflammatory response Increased macrophage infiltration and VEGF expression Induced angiogenesis	[164]
	Cu5.40-loaded heparin hydrogel	NIH-3T3 HUVEC Diabetic mice	Decreases immune cell influx and inflammatory signals at the wound site by capturing inflammatory chemokines ROS Scavenging Promote wound healing	[165]
	Lactococcus-loaded heparin poloxamer hydrogel	HUVEC Macrophages Mice	Controlled release of bioactive proteins and immune-regulating molecules Reduces inflammatory microenvironment Enhanced angiogenesis	[166]
Insulin	Insulin-encapsulated silk fibroin microparticles	HaCaT Sprague Dawley rats	Sustained release of insulin Enhanced collagen deposition and vascularization The accelerated wound closure rate	[126]
	PCL/COLL loaded with insulin chitosan nanoparticles	L929 Male Wistar rats	Enhanced blood compatibility Protection against bacterial infection Enhanced cell adhesion, growth, and migration	[126]
	Insulin-loaded Poly(vinyl alcohol)-borate hydrogels	Male Sprague Dawley rats (diabetic)	Topical insulin delivery Granulation tissue formation rate and angiogenesis accelerated The localized rise in collagen deposition	[130]
	Insulin-loaded chitosan nanoparticles	Female Wistar rats (diabetic)	Stimulated vasodilation, proliferation, leukocyte chemotaxis Accelerated epithelialization and collagen deposition	[127]
	Insulin-loaded core–shell cobalt nanoparticles	HEKa	Enhanced cell migration and wound closure rate Targeted drug delivery Highly potent for bioimaging	[131]
	Zinc insulin quantum clusters	HEKa	Enhanced cell migration and wound closure rate Targeted drug delivery Highly potent for bioimaging	[15]
	Insulin-loaded silver nanoparticles	HEKa Male Wistar rats	Regulation of balance between pro and anti-inflammatory cytokines Enhanced cell migration and wound closure rate Targeted drug delivery	[14]
	Insulin nickel quantum clusters	HEK-293	Enhanced cell migration and wound closure rate Targeted drug delivery Highly potent for bioimaging in vitro and in silico lead detection	[16]
Silk Fibroin	Curcumin-loaded polycaprolactone/polyvinyl alcohol-based silk fibroin-based electrospun nanofibrous mat	Albino female mice	Antioxidant and anti-inflammatory activity Restore the skin structure and histology of tissue at a faster pace Faster wound healing	[134]
	Melanin and berberine-amalgamated silk fibroin hydrogel	NIH3T3 Wistar rat model (diabetic)	Enhanced biocompatibility Prevent oxidative stress Enhanced migration of fibroblasts, re-epithelialization, and wound closure	[135]
	*Aloe vera* gel-loaded silk fibroin formulations	Albino mice (diabetic)	Enhanced growth of collagen fibers and blood vessels Much decreased inflammation	[136]
	Silk fibroin/gelatin/hyaluronic acid-based films impregnated with boric acid	L929	Increased cell migration activity in vitro Promoted wound healing	[137]
	Silk fibroin crosslinked glycyrrhizic acid and silver hydrogel	*Staphylococcus aureus* infected wound in mice Chorioallantoic membranes	Enhanced biocompatibility and mechanical properties Antibacterial and anti-inflammatory effects Promote tissue regeneration	[138]
	Aerosolized nanopowder having neomycin and *Avicenna marina* extract loaded in silk fibroin	CRL2522	Potent increase in cell viability Enhanced antioxidant activity Excellent antibacterial activity	[139]
	Adipose-derived stem cell-seeded silk fibroin chitosan film	Male Sprague Dawley rats (diabetic)	Accelerated cell growth Differentiation of adipocyte and osteocyte enhanced Enhanced granulation tissue formation, capillary formation, re-epithelialization	[140]
	Epidermal growth factor-conjugated silk fibroin-immobilized polyurethane	NIH3T3 Rat model (normal and diabetic)	Enhanced granulation, collagen deposition, re-epithelialization Restoration of proinflammatory cytokines IL-6,8,10 No sign of infection at the wound site	[141]
	Biliverdin silk fibroin hydrogel	GL261 Male BALB/c nude mice	Enhanced anti-inflammatory effect Stimulated angiogenesis Full-thickness wound healing	[142]
	Hyaluronic acid containing silk fibroin scaffold	HUVEC Sprague Dawley rats	Enhanced cell adhesion, proliferation, and differentiation Regulation of collagen arrangement Inhibited scar formation	[143]
	Zinc oxide-loaded PLGA/silk fibroin electrospun membranes	L929 Male Sprague Dawley rats	Enhanced re-epithelialization, collagen deposition, granulation tissue formation, and angiogenesis Antioxidant and antibacterial activity	[144]
Keratin	*Avena sativa* extract-loaded konjac glucomannan-keratin hydrogel scaffold	NIH/3T3 Male Wistar rats	Exhibit desirable swelling, biocompatibility, antibacterial, and antioxidant activity Enhanced wound contraction and collagen synthesis Increased epidermis layer and blood vessel synthesis	[145]
	Chlorhexidine-loaded polysulfobetaine/keratin hydrogel	L929 Sprague Dawley rats	Reduced inflammation Enhanced collagen deposition Antioxidant and antibacterial	[169]
	Recombinant keratin nanoparticles	HaCaT Male Sprague Dawley rats	Enhanced cell migration and proliferation Improved epithelialization, vascularization, remodeling, and collagen deposition No systemic toxicity in vivo	[146]
	Chicken feather keratin hydrogel	Skin cells	Enhanced migration of keratinocytes from the boundary to the inner wound site	[147]
	Ciprofloxacin-loaded keratin hydrogel	Female Yorkshire pigs *P. aeruginosa*	Decrease in the amount of *P. aeruginosa* by 99% Collagen-rich granulation tissue and fibroblasts at the wound site Enhanced re-epithelialization and wound contraction	[148]
	Keratin hydrogel loaded with human platelet lysate	HDF	Sustained release of pro-regenerative growth factors Promoted and supported cell proliferation without causing toxicity for upto three days	[149]
	Keratin-based dressing (keragel T)	Patient with recessive dystrophic epidermolysis bullosa	Enhanced keratinocyte proliferation and migration Accelerated epithelialization, epidermal migration, and keratinocyte activation	[150]
	Nanocomposites based on polycaprolactone/polyethylene oxide loaded with hyaluronic acid and keratinin	L929	Non-toxic nature Enhanced cell proliferation and viability	[151]
Collagen	Curcumin-loaded fish scale collagen hydroxypropyl methylcellulose nanogel	Male albino Wistar rats	High stability, wound contraction ability, and safe for dermatological application	[154]
	Collagen/chitosan-based porous scaffold	HDF Male Wistar rats	Enhanced fibroblast migration, and proliferation Increased collagen deposition and synthesis of Collagen IV angiogenesis	[170]
	Mupirocin-loaded chitosan microspheres embedded in collagen scaffold	Unisex Wistar rats	Collagen deposition Fibroblast proliferation No inflammation	[171]
	Norfloxacin-loaded collagen/chitosan scaffold	Albino rats	High water uptake and retention ability Enhanced bioadhesive strength and slow biodegradation 100% drug release ability Accelerated tissue regeneration Intact epidermal and dermal structure	[155]
	Collagen scaffold	STZ-induced diabetic rat	Accelerated migration of fibroblasts and keratinocytes Enhanced angiogenetic activity Decreased inflammation	[156]
	Collagen-laminin dermal matrix impregnated with resveratrol-loaded hyaluronic acid-DPPC microparticles	Male Wistar albino rats	Excellent antioxidant activity eventually promotes wound healing Enhanced cell proliferation of fibroblasts, keratinocytes, and endothelial cells Maintain matrix integrity by acting against collagenase	[157]
	Pioglitazone-loaded collagen/chitosan scaffold	3T3-L1 Male wistar albino rats	Optimum porosity, low matrix degradation, sustained drug release Significant decrease in matrix metalloproteinases-9 Better growth of fibroblast Potent anti-inflammatory effect	[158]
	Neurotensin-loaded collagen matrix	HaCaT Raw 264.7 Male C57BL/6 Mice	Reduced inflammatory cytokine expression Decreased inflammatory filtrate and metalloproteinases Increased fibroblast migration Enhanced deposition of collagen, collagen type III, and alpha 1	[159]
Heparin	Heparin microislands	Mice HDF HDMVEC	Epidermal regeneration and revascularization in a diabetic model Downstream cell migration in vitro	[160]
	Heparin/Prussian blue-loaded nanofibers	NIH3T3 C57/BL mice	Decreases intracellular ROS level Prevent cells from ROS-mediated apoptosis Accelerate the inflammatory response and wound healing	[172]
	Heparin-based sericin hydrogel-encapsulated fibroblast growth factor	BALB/c mice HDF	Enhanced adhesiveness Excellent biodegradability Increased collagen deposition, vascularization and re-epithelialization	[173]
	Heparin poloxamer	ECC Female Sprague Dawley rats	Enhanced proliferation and angiogenesis of endometrial epithelial cells Inhibit cellular apoptosis in the endometrium	[161]
	Heparin mimetic alginate sulfate/polycaprolactone nanoparticles	HaCaT	Controlled drug delivery Protection of drug from degradation Enhanced cellular migration, proliferation, and matrix deposition	[162]
	Heparin-loaded chitosan nanofibers	chorionic allantoic membrane	Stimulated angiogenesis	[163]
	Heparin mimetic peptide nanofibers	Male Sprague Dawley rats (Diabetic)	Promote angiogenesis, re-epithelialization, and inflammatory response Increased macrophage infiltration and VEGF expression Induced angiogenesis	[164]
	Cu5.40-loaded heparin hydrogel	NIH-3T3 HUVEC Diabetic mice	Decreases immune cell influx and inflammatory signals at the wound site by capturing inflammatory chemokines ROS Scavenging Promote wound healing	[165]
	Lactococcus-loaded heparin poloxamer hydrogel	HUVEC Macrophages Mice	Controlled release of bioactive proteins and immune-regulating molecules Reduces inflammatory microenvironment Enhanced angiogenesis	[166]

## Growth factors and growth regulators in healing wounds

Growth factors, like proteins, play a crucial role in wound healing. These physiologically active proteins bind to a specific receptor and stimulate molecular mechanisms for various cell functions, supporting cell proliferation, differentiation, migration, metabolism, and wound healing [174]. FGF is known to upregulate the activation of MEK-P, which in turn activates the NF-kβP50/P50 and inhibits NF-kβP50/P65 expression responsible for the activation of pro-inflammatory cytokines such as IL-1β, IL-6, IL-12, and TNF-α [38, 175]. FGF also upregulates the expression of Akt, which activates eNOS, STAT3, PI3K, and MMP, responsible for tissue growth and angiogenesis [176]. Nguyen et al. in 2017 prepared FGF-loaded carboxyl methyl chitosan (CMCS) nanoparticles (FGF-CMCS NPS) using an ionic gelation method for biological applications and FGF-2 remains protected from degradation of trypsin and thus act as an efficient way of FGF2 delivery at wound site for in vivo applications [177] (Fig. 8L). Butko et al. in 2016 loaded fibroblast growth factor (bFGF) into N-succinyl-chitosan and chitosan/TPP to prepare its nanoformulation with 60% encapsulating efficiency. The bFGF stimulates the transition from inflammation to cell proliferation, remodeling, and wound recovery [178]. Also, Cetin et al., in 2007, reported bFGF (basic fibroblast growth factor; belongs to the FGF family), and a pleiotropic growth factor-loaded chitosan particles (bFGF-CNPs) with 27.388% encapsulation efficiency and the particles were found to be unaffected in their structure by any changes in release parameters and encapsulation procedure, thus maintaining their stability [179] (Fig. 8M).

Losi et al. [180] prepared bFGF and vascular endothelial growth factor (VEGF)-loaded poly lactic-co-glycolic acid (PLGA) nanoparticles (VEGF-PLGA NPs), which had a significant effect on cell division, cell proliferation, collagen deposition, re-epithelization, and helped in wound closure in comparison with controls in addition to angiogenesis (Fig. 8N). VEGF activates PI3K, Akt, and eNOS to support cell growth and angiogenesis [181]. Further, it activates JAK/STAT3, which helps in the survival of the cells and blocks the signaling of pro-inflammatory cytokines STAT1, SOCS, etc. [182]. Chereddy et al. [183] treated wounds with VEGF-loaded PLGA nanoparticles, which showed an enhancement in collagen deposition that helps in re-epithelialization, angiogenesis, and complete recovery of the injury in 28 days in comparison with the free GFs. Murphy et al. [184] synthesized VEGF-encapsulated PLGA particles (VEGF-PLGA) through leaching and observed 70% recovery within 12 days and found to enhance the wound-healing capacity at a faster pace. Mohandas et al. [185] prepared fibrin nanoparticles for VEGF loading to help improve the in vitro and in vivo angiogenesis, ultimately leading to enhanced healing effect due to blood vessel formation (Fig. 8O). An in vitro transcription (IVT) method was used to develop VEGF-A mRNA which are encapsulated for delivery into ionizable lipid-mediated nanoparticles using microfluidic method and are found to be very effective in enhancing the proliferation of cells along with efficient delivery of mRNA, are found to be non-toxic in nature and used for diabetic wound healing [186]. A new chitosan-modified hydrogel having silver ions and epidermal growth factor encapsulated in nanoparticles is developed for healing the diabetic wounds and exhibited excellent collagen deposition and maturation ability, increased re-epithelialization, and optimized delivery of silver and growth factor at the wound site [187]. A polymeric path containing epidermal growth factor, GelMA hydrogel, and PHBV membranes is developed for treating the diabetic wounds. This path not only enhances the angiogenesis but also promotes proliferation and differentiation of fibroblasts, endothelial cells, and keratinocytes at the wound site [188]. Pan et al. [189] synthesized keratinocyte growth factor (KGF)-linked gold nanoparticles (KGF-AuNPs), which promotes keratinocyte proliferation; promotes wound healing through re-epithelization rather than granulation (Fig. 8P). KGF works similarly to VEGF through the KGF receptor (KGFR) [190]. The similar nanoparticles used for in vivo studies on diabetic rat models by Li et al. [191] showed the binding of KGF with KGF receptors. They enhanced the collagen-I level, TGF-β1, and alpha-smooth actin (α-SMA) that assists in wound healing. Muhamed et al., in 2019, synthesized KGF-loaded nanoparticles (KGF-F NPs) using fibrin which enhanced in vivo migration of cells and wound closure [192, 193] (Fig. 8Q). Like other growth factors, EGF also shifts the equilibrium toward the anti-inflammatory cytokines by activating PI3K/Akt, mTOR, MEK-P, and STAT 3, etc. [194]. Zhang et al. [195] prepared carboxymethyl chitosan (CC) nanoparticles by hydrophobic conjugation of linoleic acid with the carboxymethyl chitosan and loaded them with recombinant human epidermal growth factor (rh-EGF-CC NPs) (Fig. 8R). Controlled release of the loaded unstable growth factor showed the wound-healing effect when tested in vitro and in vivo. Even in chronic wounds, CC showed more inflammation recovery and healing efficiency than free rh-EGF. Gainza et al. [196] prepared a similar SLN particles nanocage by emulsification ultrasonication method for loading rh-EGF that showed significant wound healing compared to the free rh-EGF. In chronic wound, rh-EGF-loaded nanoparticles promote the proliferation of cells, reduce inflammation, and help in re-epithelization and wound healing. Chu et al. [197] used identical rh-EGF-loaded SLN particles (rh-EGF-SLNPs) prepared using the double-emulsion method (Fig. 8S). The growth factor-loaded particles enhance fibroblast proliferation and wound healing in diabetic rat models. Rajam et al. [198] prepared EGF and FGF encapsulated inside chitosan tpp nanoparticles having 83% and 84% release capacity, respectively, up to 35 days.

Xie et al. [199] used two growth factors, platelet-derived growth factor (PDGF-BB) (Fig. 2J) and VEGF; VEGF loaded within the nanofibers and PDGF-BB inside the PLGA nanoparticles. It accelerated wound regeneration, tissue remodeling, and collagen deposition with platelet-derived and VEGF-stimulated angiogenesis. Circolo et al. [200] showed that PDGF decreases the expression of TNF-α and IL-1 inflammatory cytokine. Piran et al. prepared electro-spun embedded chitosan nanoparticles for the controlled release of PDGF-BB (PDGF-BB CNPs) at the site of the wound (Fig. 8T). PDGF-BB showed significant changes in the chemotactic behavior of the cells, induced the proliferation of the fibroblast cells and neutrophils, caused the migration of the cells at the wound site, and helped in wound closure [201]. On the basis of all the instances mentioned above regarding the utilization of growth factors and growth regulators as potent stimulators of wound healing by regulating cellular proliferation, migration, and differentiation, more research needs to be done to enhance their stability, absorption rate, efficacy, target specificity, biocompatibility, and growth-promoting abilities to yield better outcomes in the near future. The mechanism of action of these growth factors is well known to the scientific community, but their application in preclinical and clinical trials is still lacking despite their wonderful features. Moreover, in recent years the number of patients suffering from intractable wounds, including diabetic ulcers, foot ulcers, etc., is increasing drastically, and to eliminate these ailments, research in the field of understanding the role of growth factors for optimizing the wound surroundings for treatment will be an exciting area of interest.

## Conclusion and future directions

Wound healing is a complex process involving several phases that occur simultaneously to promote faster recovery and prevent infection. Nanoformulations involving inorganic, organic, or biological precursors have been developed to make the formulations for wound healing biocompatible, cost-effective, and efficient. Protein and growth factors are commonly used due to their unique properties. They control inflammation at the wound site through distinct signaling pathways, leading to fast recovery. Protein-based nanoformulations can be entrapped inside the particle, protein-embedded particles, or loaded on the surface of the particles. Further, the incorporation of proteins into nanoformulations promotes the stability and activity of the proteins by preventing their degradation under unfavorable conditions. These formulations control the release of proteins for a longer time, hence enhancing efficacy and effect at the wound site. They also assist in targeted drug delivery and enhance solubility and biocompatibility. The kinetic and thermodynamic behavior of proteins and growth factors based on particles plays a critical role in timely wound healing without complications such as infection. An appropriate protein-based healing system in the form of an ointment, dressing, scaffold, hydrogel, film, powder, or electro-spun fibers can be utilized depending on the wound's requirements. Protein-based particles offer many advantages, including biocompatibility, efficiency, size, structure, easy availability, low production cost, and high biological efficiency. They also exhibit bioactivity, biodegradability, non-toxicity, enhanced re-epithelialization, cell growth, wound contraction, better repair, infection control, and antioxidant abilities.

The demand for protein-functionalized nanomaterials is enormously increasing, and emphasis should be laid on making the synthesis process less cumbersome and more effective. The elaborative discussion of the future perspective of these formulations is very crucial as their development will revolutionize healthcare facilities in wound healing in the near future, and these should be developed by keeping in mind the specific target or the effective area in the body. These formulations have a bright future in therapeutic and theranostics, and they can be used as promising drug carriers making their delivery targeted and effective. The proteins that can be transformed into scaffolds or fibers have a wider potency to act as a carrier of different therapeutics, including drugs, dyes, and inorganic and small organic moieties, thus diversifying their potential applications. In the future, these nanoformulations are expected to play a significant role in developing personalized medicine for wound healing due to the advances in technology, which makes it possible to customize the formulations based on the individual patient's needs and the specific characteristics of the wound and ultimately lead to more effective and efficient wound healing by reducing the time and cost of treatment. Furthermore, the commercial potential of protein-based nanoformulations is enormous. With the increasing prevalence of chronic wounds, such as diabetic foot ulcers and pressure ulcers, the demand for effective and efficient wound-healing treatments is rising, and protein-functionalized nanoformulations have the potential to become a widely used treatment option due to their efficacy, safety, and cost-effectiveness. Several companies have already entered the protein-based nanoformulations market, and many more are expected to follow in the near future. The market is expected to grow rapidly, and the global wound care market is estimated to reach USD 24.8 billion by 2026. Developing new and innovative protein-based nanoformulations could help drive this growth further.

In conclusion, protein-based nanoformulations have shown immense potential in wound healing and have a bright future in both medical and commercial applications. Further research and development in this area are expected to lead to the development of more effective and efficient wound-healing treatments, improving the quality of life for millions of people worldwide.

## Data Availability

Not applicable.
